# Versatile Catalytic Hydrogenation Using A Simple Tin(IV) Lewis Acid

**DOI:** 10.1002/anie.201606639

**Published:** 2016-10-24

**Authors:** Daniel J. Scott, Nicholas A. Phillips, Joshua S. Sapsford, Arron C. Deacy, Matthew J. Fuchter, Andrew E. Ashley

**Affiliations:** ^1^ Department of ChemistryImperial College LondonLondonSW7 2AZUK

**Keywords:** catalysis, frustrated Lewis pairs, hydrogenation, stannylium, tin

## Abstract

Despite the rapid development of frustrated Lewis pair (FLP) chemistry over the last ten years, its application in catalytic hydrogenations remains dependent on a narrow family of structurally similar early main‐group Lewis acids (LAs), inevitably placing limitations on reactivity, sensitivity and substrate scope. Herein we describe the FLP‐mediated H_2_ activation and catalytic hydrogenation activity of the alternative LA iPr_3_SnOTf, which acts as a surrogate for the trialkylstannylium ion iPr_3_Sn^+^, and is rapidly and easily prepared from simple, inexpensive starting materials. This highly thermally robust LA is found to be competent in the hydrogenation of a number of different unsaturated functional groups (which is unique to date for main‐group FLP LAs not based on boron), and also displays a remarkable tolerance to moisture.

Since the formalization of the concept within the last decade, great attention has been focused on the development and study of frustrated Lewis pairs (FLPs): Lewis acid (LA) and base (LB) combinations that fail to form the classically expected strong adduct, typically because it is sterically precluded.^1^ The resulting combined reactivity has been found to lead to a range of novel bond activation reactions that do not require the involvement of a transition metal (TM).^2^ Of particular interest has been the activation and cleavage of H_2_, which has allowed the development of the first general methodology for TM‐free catalytic hydrogenation.^3^

Computational investigations have suggested that the primary requirements for successful activation of H_2_ by an FLP are a sufficient cumulative LA/LB strength, and a suitable steric profile.^4^ One appealing aspect of FLP chemistry is therefore the generality of the concept; indeed, FLP‐type reactions have been identified for a broad spectrum of LAs and LBs.^2^, ^5^ Nevertheless inspection of the literature reveals that, despite the apparent breadth of the field, investigations into TM‐free FLP‐catalyzed *hydrogenation* have focused overwhelmingly on a very narrow range of LAs; thus far this has exclusively been achieved using B‐based acceptors^6^ [predominantly (fluoroaryl)borane derivatives, of which B(C_6_F_5_)_3_ is prevalent],^7^ with the exception of a single report using P‐based LAs (for a limited range of activated olefins).^8^ This constrained focus is far from ideal, as examining and developing a wider variety of LAs can be expected to produce novel FLP‐catalyzed protocols that display different substrate scope and/or more favorable functional group tolerance.^9^ For example, the application of highly Lewis acidic boranes to the FLP‐catalyzed hydrogenation of organic carbonyls has been notably challenging: whilst stoichiometric reductions were reported as early as 2007,^9^ it took until 2014 until catalytic protocols were developed.^10^ This difficulty can be attributed to the strength of the interaction between the alcohol (ROH) products and the LAs, which renders the LA⋅ROH adducts strongly acidic [cf. H_2_O⋅B(C_6_F_5_)_3_; p*K*_a_=8.4 (MeCN), <1 (H_2_O, est.)];^11^ consequently, these adducts are fundamentally incompatible with the moderately strong N/P‐centered LBs typically incorporated into active FLP catalysts. Ultimately, turnover can only be achieved when such LBs are strictly excluded, due to the necessarily highly Brønsted acidic media [for example, protonated ethers, p*K*_a_(H_2_O)≪0].^10^, ^12^

Based on the above, we were motivated to investigate FLPs based on heavier p‐block LAs, which have thus far attracted scant attention for use in FLP applications.^13^ Specifically, our interest was drawn to stannylium ion “R_3_Sn^+^” (R=alkyl) LAs;^14^ these are isolobal with Ar_3_B species commonly employed in FLP chemistry, and have been calculated to possess similar hydride ion affinities (Δ*G* ${}_{H{}^{-}}$ =65.83 and 64.95 kcal mol^−1^ for *n*Bu_3_Sn‐H and [H‐B(C_6_F_5_)_3_]^−^ respectively),^15^ suggesting that they ought to demonstrate comparable reactivity in FLP H_2_ activation and hydrogenation reactions. Furthermore, C=O reductions by R_3_SnH in protic media are well known to occur via ionic hydride transfer.^16^ Crucially, however, these LAs interact only much more weakly with hydroxylic species [for example, (*n*Bu_3_Sn⋅*x* H_2_O)^+^; p*K*_a_(H_2_O)=6.25].^17^

Manners et al. have previously investigated the use of *n*Bu_3_SnOTf (an *n*Bu_3_Sn^+^ equivalent; Tf=CF_3_SO_2_) as a LA partner in FLP chemistry,13a but reported that it was not capable of activating H_2_ when combined with the strong amine base TMP (2,2,6,6‐tetramethylpiperidine) at 50 °C, whereas the B(C_6_F_5_)_3_/TMP FLP readily cleaves H_2_, even at room temperature;^18^ this result was attributed to the poorer electrophilicity of the Sn compound, and it is evident that the Sn–OTf interaction is strong enough to substantially reduce the Lewis acidity of the *n*Bu_3_Sn^+^ fragment.

We envisioned that it should be possible to increase the Lewis acidity, to the threshold necessary for favorable H_2_ heterolysis, by simply increasing the size of the alkyl groups on Sn, thereby increasing the degree of “internal frustration”^19^ between the R_3_Sn^+^ and TfO^−^ moieties. To this end, we targeted the bulkier trialkylstannyl compound *i*Pr_3_SnOTf ([**1**]OTf), which was readily prepared via reaction of excess *i*PrMgCl and SnCl_4_ to generate *i*Pr_4_Sn, followed by facile protodealkylation with HOTf (Scheme 1). This straightforward and inexpensive two‐step procedure furnishes pure [**1**]OTf in good yield (42 %, 2 steps), and can easily be performed on a multi‐gram scale. [**1**]OTf is a white solid that shows moderate solubility in polar halogenated solvents and its ^119^Sn{^1^H} spectrum shows a single broad resonance at *δ*=156 ppm (Δ*v*_1/2_=130 Hz, CDCl_3_). The high chemical shift is consistent with significant stannylium ion character, although it is considerably upfield of the value reported for [*n*Bu_3_Sn][CB_11_Me_12_] (*δ*=454 ppm), which displays the least coordinated trialkylstannylium core to date.^20^ Gutmann–Beckett Lewis acidity measurements support this conclusion,^21^ indicating increased electrophilicity in comparison with *n*Bu_3_SnOTf, although still lower than B(C_6_F_5_)_3_ 22 {AN=64.2 *n*Bu_3_SnOTf; 68.0 [**1**]OTf; 78.1 B(C_6_F_5_)_3_}. [**1**]OTf has also been characterized by ^1^H, ^13^C and ^19^F NMR spectroscopy, MS and elemental analysis (see the Supporting Information (SI)).

**Scheme 1:**
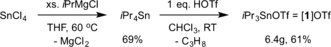
Synthesis of [**1**]OTf.

Addition of DABCO (1,4‐diazabicyclo[2.2.2]octane) to [**1**]OTf (1:1) leads to an upfield shift in the ^119^Sn{^1^H} resonance (which remains similarly broad) to 39 ppm, consistent with a donor–acceptor interaction. However, the corresponding ^1^H NMR spectrum shows only a single resonance for the DABCO protons, suggesting rapid exchange between an adduct and FLP. Admission of H_2_ (4 bar) leads to the appearance of resonances in the room temperature ^1^H [5.12 ppm, Sn*H*, ^1^ *J*(^119^Sn/^117^Sn‐^1^H)=1471/1405 Hz; 10.93 ppm, N*H*] and ^119^Sn{^1^H} (−46 ppm) NMR spectra, that are consistent with formation of *i*Pr_3_SnH ([**1**]H) and DABCO⋅HOTf, and hence H_2_ heterolysis by the N/Sn Lewis pair. Further, conclusive proof for H_2_ activation is provided by replacing H_2_ with D_2_, which causes the new ^119^Sn{^1^H} resonance to split into a triplet [1:1:1, ^1^ *J*(^119^Sn‐^2^H)=226 Hz], and the new resonances in the ^1^H NMR spectrum to be replaced by equivalent signals in the ^2^H spectrum. This represents the first example of FLP H_2_ activation using a LA based on Sn, or any p‐block element beyond the 3^rd^ row of the periodic table.

Having demonstrated H_2_ activation, our focus shifted to achieving catalytic hydrogenation using [**1**]OTf. Gratifyingly, heating the archetypal FLP substrates PhCH=N*t*Bu (**2 a**) and PhC(Me)=N*t*Bu (**2 b**) with 10 mol % [**1**]OTf to 120 °C under H_2_ (10 bar) led to conversion to the respective amines (**3 a** and **3 b**; Table 1, entries 1 and 2). Conversely, the *N*‐phenyl analogue PhCH=NPh (**2 c**) is reduced far less effectively (Table 1, entry 3), which is attributed to the reduced basicity of both the imine and amine product, which makes H_2_ activation less favorable. Consistent with this interpretation, addition of 2,4,6‐collidine [Col; p*K*_a_(MeCN)=14.98]^23^ as an auxiliary base leads to a dramatic improvement in performance (Table 1, entry 4), and also allows for reduction of the related ketimine PhC(Me)=NPh (**2 d**; Table 1, entry 5), and even PhCH=NTs (**2 e**; Ts=O_2_SC_6_H_4_Me, 4‐toluenesulfonyl), although the latter reaction is appreciably slower, presumably as the substrate is less basic still (Table 1, entry 6). Notably, the bromoaryl imine **2 f** also undergoes efficient C=N hydrogenation (Table 1, entry 7); no evidence of hydrodebromination is observed during this reaction (no NMR resonances attributable to **2 a**/**3 a**, [**1**]Br or [**1**]_2_),^24^ supporting the idea that radical Sn species do not appear to be involved in this reaction. Accordingly, we propose that hydrogenation occurs via a polar mechanism analogous to that for related borane‐catalyzed systems:1d,1e, 25 H_2_ activation by an FLP consisting of [**1**]OTf/imine is followed by hydride transfer and release of amine at elevated temperature (Figure S15). This is further supported by the observation that pre‐formed **2 a**⋅HOTf is rapidly reduced by [**1**]H even at RT,^26^ whereas the equivalent reactions with unprotonated **2 a**, either alone or in the presence of [**1**]OTf, do not lead to significant reduction at 120 °C (see SI). Interestingly, there is evidence for autocatalysis during the course of the reaction (16 % conversion observed after 3 h, 60 % after 6 h); comparable observations have been made by Paradies et al. for imine hydrogenations catalyzed by B(2,6‐F_2_C_6_H_3_)_3_, and are attributed to the increased basicity of the product amines, relative to the imine substrate, rendering H_2_ activation more favorable as more product is formed.^25^

**Table 1:** [**1**]OTf‐catalyzed hydrogenation of imines. 
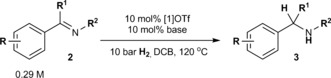

Entry^[a]^	Substrate	R	R^1^	R^2^	Base	*t* [h]	Conversion [%]^[b]^
1	**2 a**	H	H	*t*Bu	–	12	97
2	**2 b**	H	Me	*t*Bu	–	16	85
3	**2 c**	H	H	Ph	–	16	4
4	**2 c**	H	H	Ph	Col	24	>99
5	**2 d**	H	Me	Ph	Col	32	96
6	**2 e**	H	H	Ts	Col	80	65
7	**2 f**	4‐Br	H	*t*Bu	Col	16	96

[a] 10 bar refers to initial pressure at RT. [b] Conversions determined by ^1^H NMR spectroscopic analysis (see the SI).

Following success in the hydrogenation of imines, we were interested to see whether [**1**]OTf might also be capable of mediating the hydrogenation of closely related carbonyl compounds. Satisfyingly, when acetone (**4 a**) is exposed to reaction conditions similar to those used to hydrogenate **2 c** catalytic conversion to 2‐propanol (**5 a**) is observed (Table 2, entry 1). Whilst the reaction at 120 °C is somewhat slow, at 180 °C near‐quantitative conversion can be observed within 32 h (Table 2, entry 2). Significantly, no evidence of catalyst decomposition is observed in this homogeneous reaction, either by ^1^H or ^119^Sn{^1^H} NMR spectroscopy,^27^ in comparison with analogous FLP protocols mediated by B(C_6_F_5_)_3_.1f, 28 To the best of our knowledge this is the first example of a catalytically active FLP system capable of tolerating such conditions without degradation, and illustrates the impressive thermal stability of [**1**]OTf, which enables the use of more forcing conditions in order to achieve an improved rate of turnover. As well as **4 a**, other aliphatic and aromatic ketones and aldehydes (**4 b**—**d**) can be reduced under these conditions (Table 2, entries 3–5). In the case of acetophenone (**4 b**), ^1^H NMR spectroscopic analysis indicates formation of the expected alcohol **5 b**, in addition to smaller quantities of styrene (**6**) and α‐methylbenzyl ether (**7**). Similar side‐reactions were observed in our previous attempts to reduce **4 b** using B(C_6_F_5_)_3_ in 1,4‐dioxane,10b, 12c but in those cases this led to severe reductions in conversion and rate of turnover.

**Table 2:** [**1**]OTf‐catalyzed hydrogenation of ketones and aldehydes. 
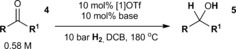

Entry^[a]^	Substrate	R	R^1^	Base	*t* [h]	Conversion [%]^[b]^
1^[c]^	**4 a**	Me	Me	Col	96	78
2^[d]^	**4 a**	Me	Me	Col	32	97
3^[d]^	**4 b**	Ph	Me	Col	48	91^[f]^
4^[d]^	**4 c**	*t*Bu	H	Col	48	79
5^[d]^	**4 d**	2,6‐Cl_2_C_6_H_3_	H	Col	32	91
6	**4 a**	Me	Me	Col	16	57
7	**4 a**	Me	Me	Lut	16	48
8	**4 a**	Me	Me	DABCO	16	14
9	**4 a**	Me	Me	[**1**]O*i*Pr^[e]^	16	32
10^[g]^	**4 a**	Me	Me	Col	32	95

[a] 10 bar refers to initial pressure at RT. [b] Conversions determined by ^1^H NMR spectroscopic analysis (see the SI). [c] Reaction run at 120 °C, repressurized after 48 h. [d] Repressurized at 16 h intervals. [e] Generated in situ from [**1**]H and **4 a** (see the SI). [f] Based on consumption of **4 b**; reaction produces **5 b** in addition to **6** and **7** as side‐products in a ca. 74:18:8 molar ratio (see the SI). [g] Using undried reagents, solvent and catalyst (see the SI).

The ease and speed with which it was possible to apply this system to carbonyl hydrogenation stands in contrast to the extended period of development required before more conventional B‐based FLPs were successfully used in this transformation.10a,10b It is also noteworthy that the [**1**]OTf‐catalyzed reaction can proceed using a rather conventional, moderately‐strong, N‐centered LB, which again contrasts with B‐based systems and is consistent with less acidic adducts forming between the product alcohols (e.g. **5 a**) and [**1**]OTf. The choice of LB is important to the outcome of the hydrogenation of **4 a** (Table 2, entries 6–8), with inferior results obtained using either a weaker or stronger base [2,6‐lutidine (Lut), DABCO; p*K*_a_(MeCN)=14.13, 18.29].^29^

Given the low Brønsted basicity of **4 a** we propose a slightly different mechanism for its reduction than for **2 a**,^30^ with the substrate activated by [**1**]^+^ rather than via H‐bonding to [Col‐H]^+^ (Scheme 2 a).16b Evidence for this comes from the significantly upfield‐shifted ^119^Sn{^1^H} NMR resonance (*δ*=92 ppm) observed upon addition of **4 a** (10 equiv.) to [**1**]OTf, indicative of Sn−O binding.^31^ A proposed subsequent H^−^ transfer from [**1**]H to adduct {[**1**]⋅**4 a**}OTf, to form [**1**]O*i*Pr and regenerate [**1**]OTf, is supported by the observation that [**1**]H is capable of reducing **4 a** in the presence of [**1**]OTf even at RT, whereas no appreciable conversion is observed in its absence either at RT or 120 °C. Conversely, if [**1**]OTf is replaced by Col⋅HOTf, only slow release of H_2_ is observed at RT.^32^ In order for the final H^+^ transfer step to occur efficiently it should be recognized that Col and [**1**]O*i*Pr must be comparable in base strength and, therefore, it may be envisaged that once [**1**]O*i*Pr is formed in the reaction mixture, it could also activate H_2_ in conjunction with [**1**]OTf (Scheme 2 b). In fact, catalytic hydrogenation *can* be observed by substituting Col with [**1**]O*i*Pr (generated in situ from [**1**]H and **4 a**; Table 2, entry 9), thus demonstrating its competence in this role. Even so, the reduced rate of turnover in this reaction indicates that the auxiliary base does play a beneficial role beyond simply facilitating formation of some initial [**1**]O*i*Pr, presumably by rendering H_2_ activation more favorable.^33^

**Scheme 2:**
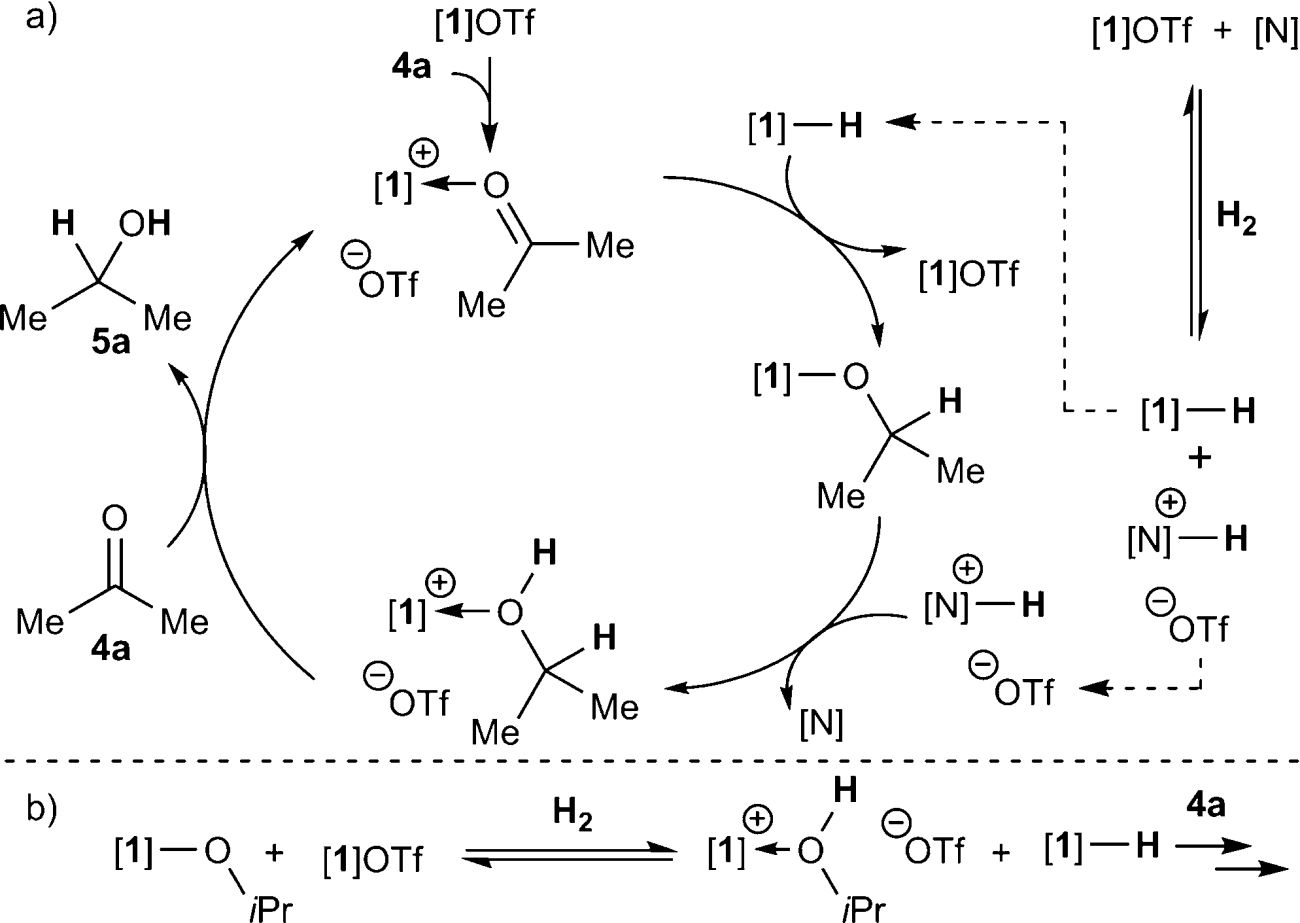
a) Proposed mechanism for catalytic hydrogenation of **4 a** using [**1**]OTf ([N]=2,4,6‐collidine) and b) alternative H_2_ activation using in situ generated [**1**]O*i*Pr.

Clear tolerance of alcohol products suggested that these reactions might also demonstrate appreciable moisture tolerance.^10^, ^12^ Remarkably, when the hydrogenation of model substrate **4 a** (chosen over an imine to avoid hydrolysis) was prepared on the open bench, with non‐anhydrous reagents and solvent, and using [**1**]OTf that had been exposed to air for 1 week, the reaction was observed to proceed without any noticeable reduction in rate (Table 2, entry 10; details in SI). This is unprecedented in FLP catalysis, where even the most tolerant of previously reported reactions have been dramatically slowed by adventitious H_2_O,^12^ and suggests a major advantage of using Sn‐based LAs.

Finally, we investigated the use of [**1**]OTf in the catalytic hydrogenation of compounds containing other unsaturated functionalities; the heteroaromatic ring of acridine, and the C=C bonds in *n*‐butyl acrylate and 1‐piperidino‐1‐cyclohexene could all be effectively reduced (yields 83–99 %), further demonstrating the versatility of this Sn^IV^ compound (Figure S33).

In summary, we have demonstrated the use of readily accessible and inexpensive *i*Pr_3_SnOTf as a main‐group LA catalyst for the hydrogenation of C=C, C=N and C=O bonds; this constitutes only the second example of an FLP hydrogenation protocol utilizing a p‐block LA not incorporating boron, and the first such example shown to be applicable to the reduction of a range of different functional groups. Despite the ubiquity of Sn in industrial catalysis this also represents, to the best of our knowledge, the first example of homogeneous catalytic hydrogenation using a Sn‐based system of any kind.^34^ Of particular interest is the ready applicability of this protocol to C=O bond hydrogenation, in a reaction that displays an unparalleled level of H_2_O tolerance. This neatly demonstrates the value of pursuing alternative FLP LAs, and can be jointly attributed to the formation of weakly acidic LA⋅ROH adducts; a thermally robust [*i*Pr_3_Sn]^+^ core, allowing access to high reaction temperatures; and the stability of the Sn−C bonds towards protolytic cleavage for example, by H_2_O. Clearly there is significant scope for variation of the triorganotin(IV) framework in “R_3_Sn^+^” species; investigations into how this affects their reactivity, functional group tolerance, and substrate scope are currently underway.

## Supporting information

As a service to our authors and readers, this journal provides supporting information supplied by the authors. Such materials are peer reviewed and may be re‐organized for online delivery, but are not copy‐edited or typeset. Technical support issues arising from supporting information (other than missing files) should be addressed to the authors.

Supplementary
