# Supplementary material for: Versatile Catalytic Hydrogenation Using A Simple Tin(IV) Lewis Acid
Source: Angew Chem Int Ed Engl. 2016 Oct 24;55(47):14738–42. doi: 10.1002/anie.201606639 (PMC5129554; doi:10.1002/anie.201606639)
Supplement: Supplementary file 1 — Supplementary [file ANIE-55-14738-s001.pdf]

Angewandte Chemie
Eine Zeitschrift der Gesellschaft Deutscher Chemiker

Supporting Information

Versatile Catalytic Hydrogenation Using A Simple Tin(IV) Lewis Acid

*Daniel J. Scott, Nicholas A. Phillips, Joshua S. Sapsford, Arron C. Deacy, Matthew J. Fuchter, and Andrew E. Ashley**

anie_201606639_sm_miscellaneous_information.pdf

Contents **Page**

1. General experimental considerations S3
2. Synthesis of [**1**]X (X = *i*Pr, OTf, H) S3
   - 2.1. Synthesis of *i*Pr_4Sn S3
   - 2.2. Synthesis of *i*Pr_3SnOTf ([**1**]OTf) S4
   - 2.3. Independent synthesis of *i*Pr_3SnH ([**1**]H) S6
3. Gutmann-Beckett Lewis acidity measurements S7
   - 3.1. General procedure S7
   - 3.2. Summary of values S7
4. H_2 activation using [**1**]OTf / base S7
   - 4.1. H_2 activation with DABCO S7
   - 4.2. D_2 activation with DABCO S9
   - 4.3. H_2 activation with lutidine S11
   - 4.4. H_2 activation with collidine S13
5. Typical procedure for hydrogenation of imines **2** catalysed by [**1**]OTf S14
6. Proposed mechanism for hydrogenation of imines **2** by [**1**]OTf S19
   - 6.1. Stoichiometric addition of [**1**]H to **2a** S19
   - 6.2. Stoichiometric addition of [**1**]H to **2a**·HOTf S19
   - 6.3. Stoichiometric addition of [**1**]H to **2a** / [**1**]OTf S20
7. Typical procedure for hydrogenation of aldehydes and ketones **4** catalysed by [**1**]OTf S20
8. Mechanistic investigations into the hydrogenation of **4a** S24
   - 8.1. Hydrogenation of **4a** catalysed by [**1**]OTf / [**1**]O*i*Pr S24
   - 8.2. Stoichiometric addition of [**1**]H to **4a** S25
   - 8.3. Stoichiometric addition of [**1**]H to **4a /** collidine·HOTf S25
   - 8.4. Stoichiometric addition of [**1**]H to **4a** / [**1**]OTf S27
   - 8.5. A note on the mechanism of carbonyl hydrogenation, and the effect of base strength S29
9. 'Open bench' hydrogenation of **4a** catalysed by [**1**]OTf S30
10. Hydrogenation of additional substrates catalysed by [**1**]OTf S31
    - 10.1. Hydrogenation of acridine S31
    - 10.2. Hydrogenation of *n*-butyl acrylate S32
    - 10.3. Hydrogenation of 1-piperidino-1-cyclohexene S33
11. References S34

1. General experimental considerations

All reactions were performed under N_2 atmosphere unless stated otherwise. All manipulations were carried out either in an MBraun Labmaster DP glovebox or by using standard Schlenk line techniques. All glassware was dried by heating to 170 °C overnight before use. All solvents were degassed and dried before use: THF was distilled under N_2 from Na / fluorenone and stored over 4 Å molecular sieves; MeOH was dried by standing over sequential batches of 3 Å molecular sieves; pentane was dried using an Innovative Technology Pure Solv™ SPS-400 and stored over K; $CHCl_3$ was dried using an Innovative Technology Pure Solv™ SPS-400 and stored over 3 Å molecular sieves; 1,2-difluorobenzene was dried by refluxing over CaH_2, distilled, and stored over 4 Å molecular sieves; 1,2-dichlorobenzene was purchased anhydrous from Sigma-Aldrich and further dried and stored over 5 Å molecular sieves; $CDCl_3$ and CD_2Cl_2 were freeze-pump-thaw degassed and dried over 4 Å molecular sieves. Imines **2b**,[1] **2d**[2] and **2f**[3] were prepared in accordance with the literature. Isopropanol was degassed and dried over 4 Å molecular sieves. Acetone was degassed, dried over B_2O_3 and distilled. Mg turnings were heated to 170 ºC overnight before use. *i*PrCl, $SnCl_4$, HOTf, $NaBH_4$, *n*Bu_3SnOTf and Et_3PO were purchased from major suppliers and used as provided. All other compounds were purchased from major suppliers: solids were dried under vacuum, while liquids were degassed and dried over 4Å molecular sieves. H_2 was purchased from BOC (research grade) and dried by passage through a Matheson Tri-Gas Weldassure™ Purifier drying column. D_2 (99.8% D) was purchased from Cambridge Isotope Laboratories and dried by standing over 3 Å molecular sieves. Elemental analysis was performed by Stephen Boyer of London Metropolitan University. NMR spectra were recorded on Bruker AV-400 and DRX-400 spectrometers. ^{1}H and ^{2}H spectra were referenced internally to residual solvent signals, while ^{19}F and $^{119}Sn\{^{1}H\}$ spectra were referenced externally to $CFCl_3$ and $SnMe_4$ respectively. Chemical shifts are stated in ppm (s = singlet, d = doublet, q = quartet, sp = septet, m = multiplet, br = broad).

Conversions were calculated by ^{1}H NMR integration, either by relative integration of product and starting material resonances (in cases where no other species were observed), or by integration relative to $SiMe_4$ added as an internal standard. In cases where the final reaction mixture was not fully homogeneous at RT, spectra were also acquired of homogeneous solutions at elevated temperature. In order to minimise any errors, integrations were performed on the most intense product/substrate resonances wherever possible, and only on signals well separated from other peaks. Typically, the intensity of a particular product resonance was compared to the intensity of the resonance for the same protons in the starting material.

2. Synthesis of [1]X (X = *i*Pr, OTf, H)

2.1. Synthesis of *i*Pr_4Sn

To a suspension of Mg turnings (5.64 g, 232 mmol) in THF (40 mL) was added dropwise a solution of *i*PrCl (21.2 mL, 232 mmol) in THF (80 mmol) at RT (maintained through use of a water bath). After stirring for 20 h the solution was filtered dropwise over 4 h onto a stirred suspension of $SnCl_4$ (13.4 g, 51.5 mmol) in THF (120 mL), which was maintained at 0 ºC through use of an ice bath. The solid residue was washed with further THF (30 mL), which was filtered across in an identical manner. The resulting suspension was heated to 60 ºC for 25 h, cooled to RT, and extracted into pentane (3 x 150 mL). The remaining work-up was performed under air: the solution was dried over $MgSO_4$ and

filtered, and the solvent removed under reduced pressure. The resulting oil was distilled (110 ºC, 1 mbar) to afford *i*Pr_4Sn as a colourless oil (10.4 g, 69 %).

^{1}H NMR (400 MHz, $CDCl_3$) δ: 1.32 [6H, d, $^3J(^1H-^1H)$ = 7.2 Hz, $^3J(^{117}Sn-^1H)$ = 29 Hz, $^3J(^{119}Sn-^1H)$ = 30 Hz, C*H_3*], 1.42-1.55 [1H, m, C*H*]. $^{119}Sn\{^1H\}$ NMR (149 Hz, $CDCl_3$) δ: -42.9 (s); these values are consistent with those previously reported in the literature[4].

2.2. Synthesis of *i*Pr_3SnOTf ([1]OTf)

To a solution of *i*Pr_4Sn (8.0 g, 27.5 mmol) in $CHCl_3$ (80 mL) was added HOTf (3.9 g, 26.2 mmol) in $CHCl_3$ (40 mL). The mixture was stirred at RT for 5 days before the solvent was removed *in vacuo* and the resulting solid washed with pentane (2 x 15 mL), affording *i*Pr_3SnOTf as a white solid (6.4 g, 61 %).

^{1}H NMR (400 MHz, $CDCl_3$) δ: 1.44 [6H, d, $^3J(^1H-^1H)$ = 7.6 Hz, $^3J(^{117}Sn-^1H)$ = 86 Hz, $^3J(^{119}Sn-^1H)$ = 90 Hz, C*H_3*], 2.07 [1H, sp, $^3J(^1H-^1H)$ = 7.6 Hz, $^2J(^{119}Sn-^1H)$ = 39 Hz, C*H*]. $^{13}C\{^1H\}$ NMR (101 MHz, $CDCl_3$) δ: 20.7 [s, $^2J(^{117/119}Sn-^{13}C)$ = 16 Hz, *C*H_3], 27.2 [s, $^1J(^{117}Sn-^{13}C)$ = 302 Hz, $^1J(^{119}Sn-^{13}C)$ = 316 Hz, *C*H], 119.0 [q, $^1J(^{19}F-^{13}C)$ = 319 Hz, *C*F_3]. ^{19}F NMR (376 MHz, $CDCl_3$) δ: -76.7 (s). $^{119}Sn\{^1H\}$ NMR (149 MHz, $CDCl_3$, 0.06 M) δ: 156 [br s, $\Delta\nu_{1/2}$ = 130 Hz]. MS (APCI) m/z: 327 (*i*$Pr_3SnOSO_2^+$), 249 (*i*Pr_3Sn^+). Anal. calcd. for $C_{10}H_{21}F_3O_3SSn$: C, 30.25; H, 5.33. Found: C, 30.08; H, 5.45.

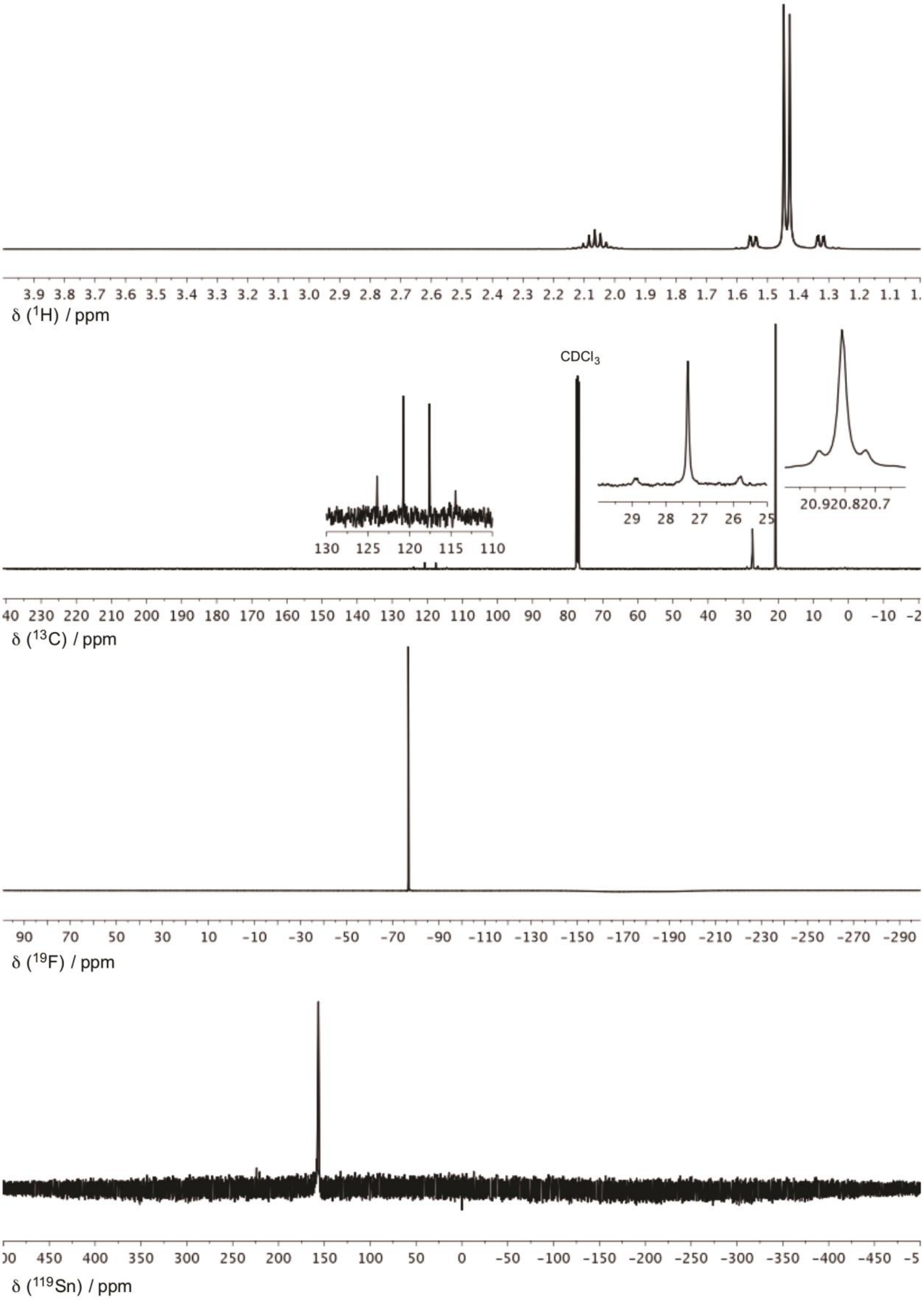

Figure S1 – ^{1}H, ^{13}C{^{1}H}, ^{19}F and ^{119}Sn{^{1}H} NMR spectra of [**1**]OTf (0.06 M in $CDCl_3$).

2.3. Independent synthesis of *i*Pr_3SnH (^{1}H)

To a solution of $NaBH_4$ (53.1 mg, 1.21 mmol) in MeOH (15 mL) was added *i*Pr_3SnOTf ([**1**]OTf, 558 mg, 1.21 mmol) in MeOH (15 mL). The solution was stirred for 3 h before cooling to -78 ºC and extracting with pentane (3 x 10 mL), which was allowed to warm to RT, dried over $MgSO_4$, and filtered. The solvent was carefully removed under reduced pressure (RT, 500 mbar) and the resultant liquid distilled (85 ºC, 10 mbar), yielding the product as a colourless liquid (76 mg, 25%).

^{1}H NMR (400 MHz, C_6D_6) δ: 1.20-1.51 [21 H, m, C*H*(C*H*$_3$)$_2$], 5.32 [1H, s, $^1J(^{117}$Sn-^{1}H) = 1408 Hz, $^1J(^{119}$Sn-^{1}H) = 1474 Hz, Sn*H*, T_1 = 36.2 s]. ^{13}C{^{1}H} NMR (101 MHz, C_6D_6) δ: 13.6 [s, *C*H], 22.7 [s, $^2J(^{117/119}$Sn-^{13}C) = 15 Hz, *C*H$_3$]. ^{119}Sn{^{1}H} NMR (149 MHz, C_6D_6) δ: –49 (s).

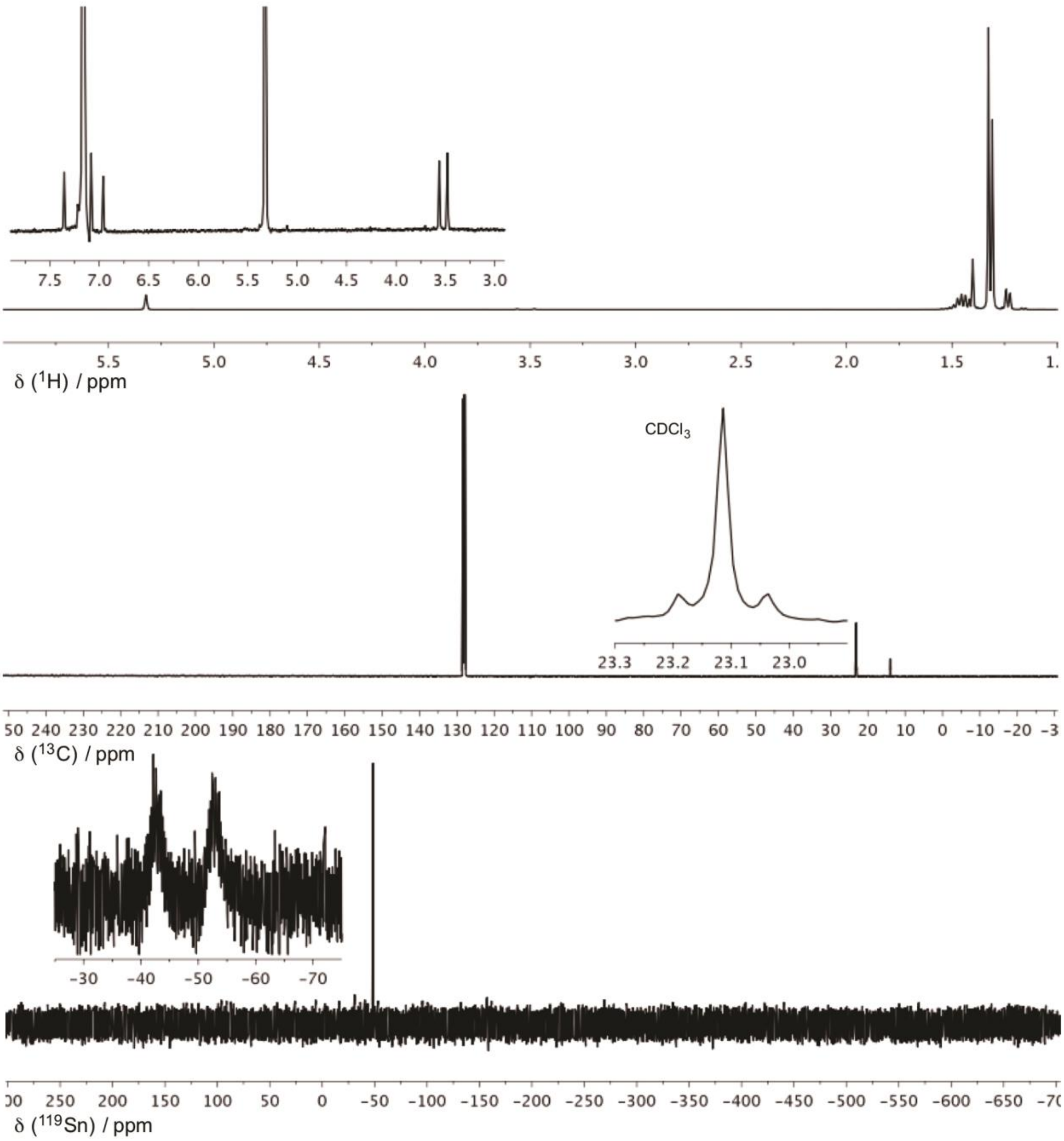

Figure S2 – ^{1}H [with inset showing $^1J(^{117/119}$Sn-^{1}H) satellites], ^{13}C{^{1}H} and ^{119}Sn{^{1}H} (with ^{119}Sn inset) NMR spectra of [**1**]H in C_6D_6.

3. Gutmann-Beckett Lewis acidity measurements

3.1. General procedure[5]

A mixture of the Lewis acid (0.06 mmol) and Et_3PO (3.6 mg, 0.02 mmol) was dissolved in CD_2Cl_2 (0.4 mL) in a NMR tube fitted with a J. Young's valve. A capillary insert containing 1 M Et_3PO in CD_2Cl_2 was also added to act as a $^{31}P\{^1H\}$ reference. Based on the $^{31}P\{^1H\}$ chemical shift of the resulting Et_3PO adduct, the acceptor number was calculated according to the formula of Beckett *et al.*:[6] AN = [δ(sample) / ppm – 41.0] x 2.22.

3.2. Summary of values

Lewis acid	$\delta(^{31}P)$ / ppm	AN
$B(C_6F_5)_3$[7]	77.0	78.1
nBu_3SnOTf	70.0	64.2
iPr_3SnOTf ([**1**]OTf)	71.7	68.0

4. H_2 activation using [1]OTf / base

As described in our main manuscript, [**1**]OTf is capable of activating H_2 in combination with DABCO. We have also demonstrated that H_2 activation is capable using the weaker bases lutidine and collidine (as evidenced by the formation of $[N]H^+$ and [**1**]H resonances in ^1H and $^{119}Sn\{^1H\}$ NMR spectra; see below). H_2 activation is significantly less favoured with these bases, even at higher pressure, and is less favoured for lutidine than collidine. These observations are consistent with the lower basicity of lutidine than collidine, and of collidine than DABCO.

4.1. H_2 activation with DABCO

[**1**]OTf (15.9 mg, 0.04 mmol) and DABCO (4.5 mg, 0.04 mmol) were dissolved in 1,2-difluorobenzene (0.7 mL) in an NMR tube fitted with a J. Young's valve, to which was also added a capillary insert containing PPh_3 in C_6D_6 (to provide a lock and reference). Initial ^1H and $^{119}Sn\{^1H\}$ NMR spectra were recorded, H_2 was added *via* a freeze-pump-thaw method (1 bar at -196 ºC, *ca.* 4 bar at RT), and the sample allowed to stand for 2 days (with occasional agitation) before being re-analysed. Note that due to slow relaxation of the Sn*H* resonance (*ca.* 15 s, measured *in situ*), the final ^1H NMR spectrum was recorded using an extended delay of 100s.

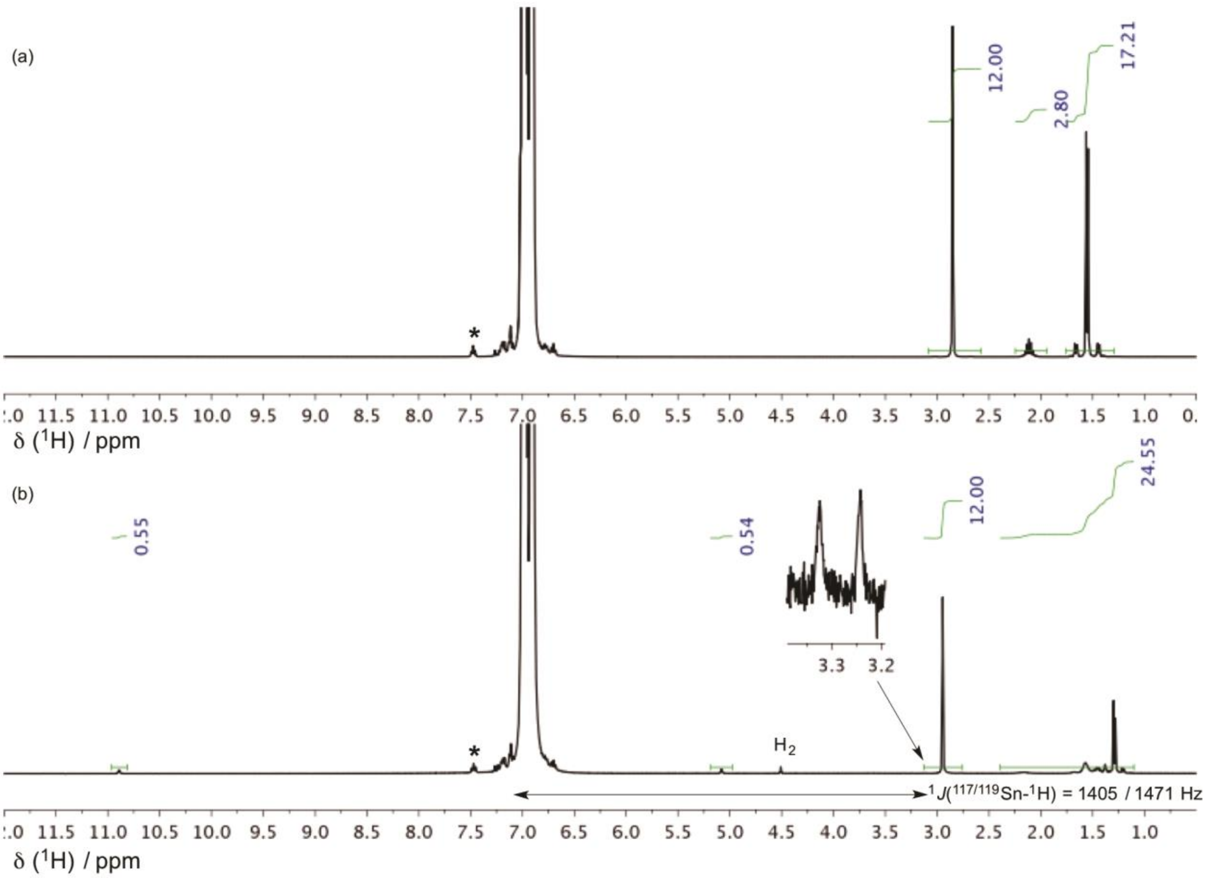

Figure S3 – ^{1}H NMR spectra of [**1**]OTf / DABCO in 1,2-difluorobenzene before (a) and after (b) admission and activation of H_2 (4 bar). * = PPh_3 in capillary insert. Inset shows upfield $^{1}J(^{117/119}$Sn-^{1}H) satellites (downfield satellites obscured by solvent peaks).

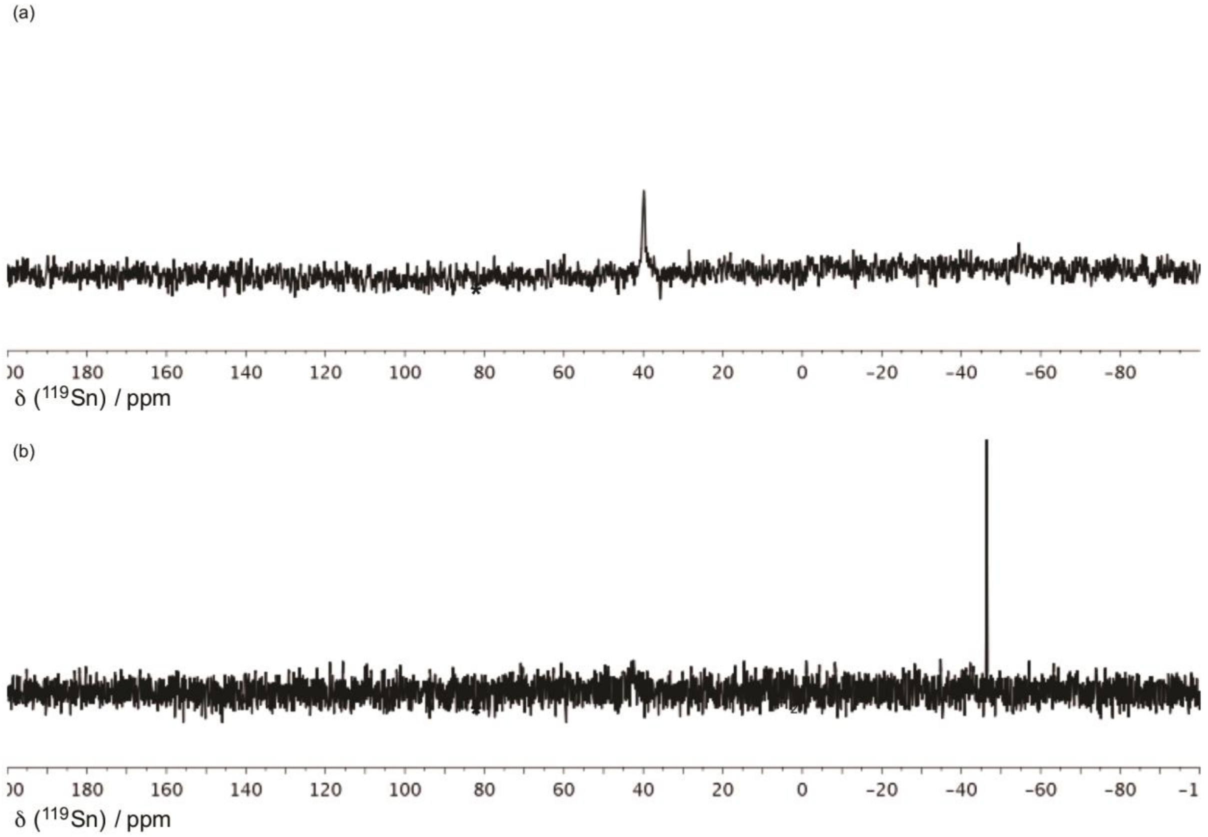

Figure S4 – ^{119}Sn{^{1}H} NMR spectra of [**1**]OTf / DABCO in 1,2-difluorobenzene before (a) and after (b) admission and activation of H_2 (4 bar).

4.2. D_2 activation with DABCO

[**1**]OTf (15.9 mg, 0.04 mmol) and DABCO (4.5 mg, 0.04 mmol) were dissolved in 1,2-difluorobenzene (0.7 mL) in an NMR tube fitted with a J. Young's valve. Initial ^{1}H, ^{2}H and ^{119}Sn{^{1}H} NMR spectra were recorded, D_2 was added through use of a Toepler pump (*ca*. 2 bar), and the sample allowed to stand for 2 days (with occasional agitation) to allow it to reach equilibrium before being re-analysed.

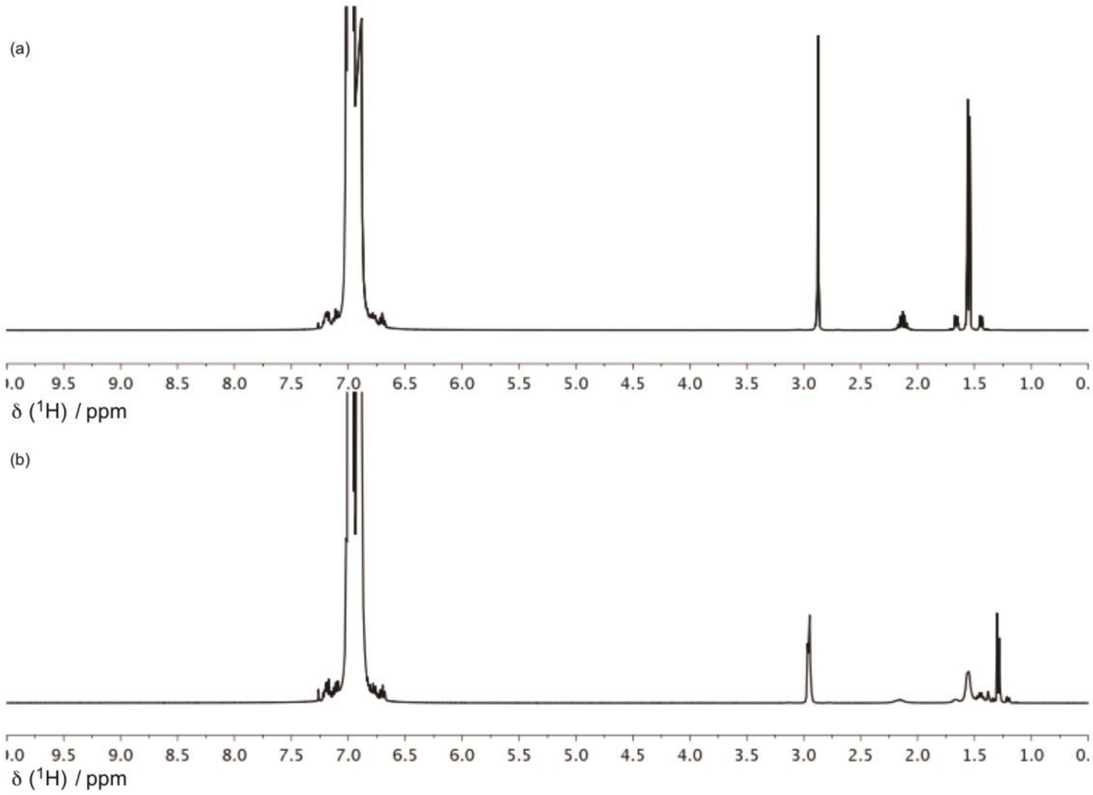

Figure S5 – ^{1}H NMR spectra of [**1**]OTf / DABCO in 1,2-difluorobenzene before (a) and after (b) admission and activation of D_2 (2 bar).

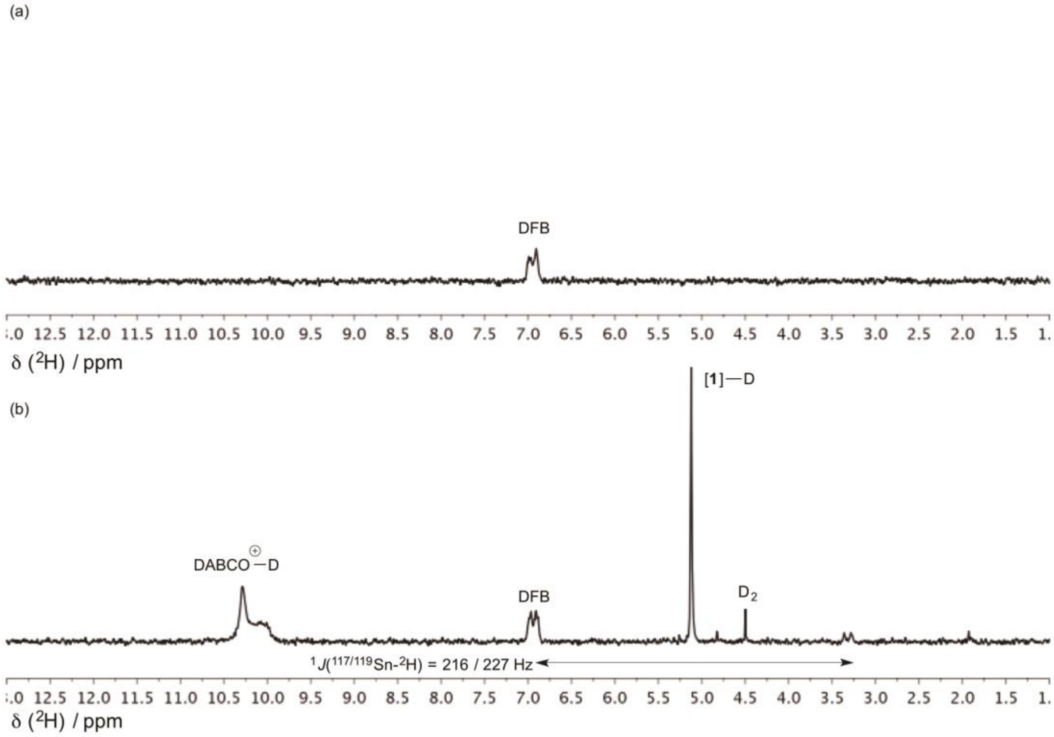

Figure S6 – ^{2}H NMR spectra of [**1**]OTf / DABCO in 1,2-difluorobenzene before (a) and after (b) admission and activation of D_2 (2 bar). Inset shows upfield $^{1}J(^{117/119}$Sn-^{2}H) satellites (downfield satellites obscured by solvent peaks).

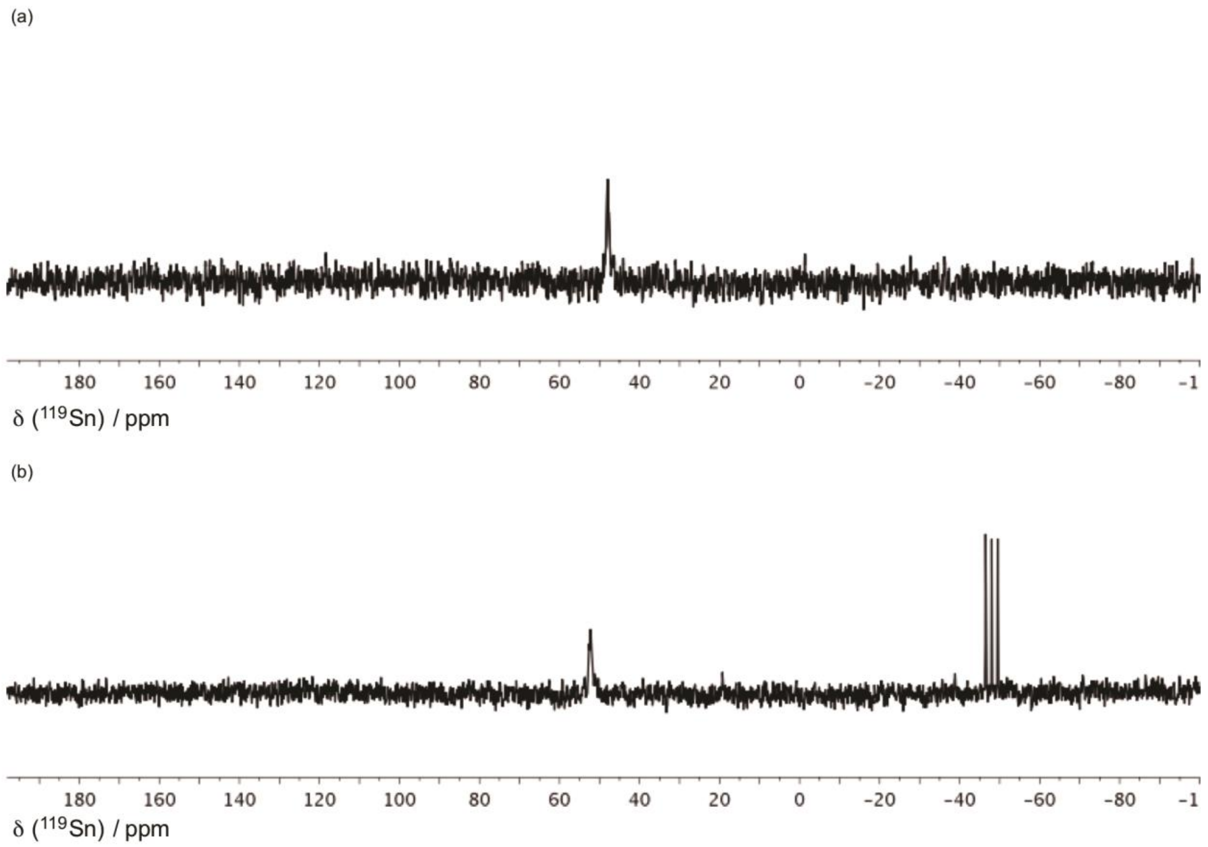

Figure S7 – ^{119}Sn{^{1}H} NMR spectra of [**1**]OTf / DABCO in 1,2-difluorobenzene before (a) and after (b) admission and activation of D_2 (2 bar).

4.3. H_2 activation with lutidine

[**1**]OTf (15.9 mg, 0.04 mmol) and lutidine (4.7 μL, 0.04 mmol) were dissolved in 1,2-difluorobenzene (0.7 mL) in a Wilmad high pressure NMR tube fitted with a PV-ANV PTFE valve. H_2 was admitted up to a pressure of 10 bar (at RT), and the sample was allowed to stand for 20 hours (with occasional agitation) before being re-analysed. Note that due to slow relaxation of the Sn*H* resonance (*ca.* 14 s, measured *in situ*), the final ^{1}H NMR spectrum was recorded using an extended delay of 100s.

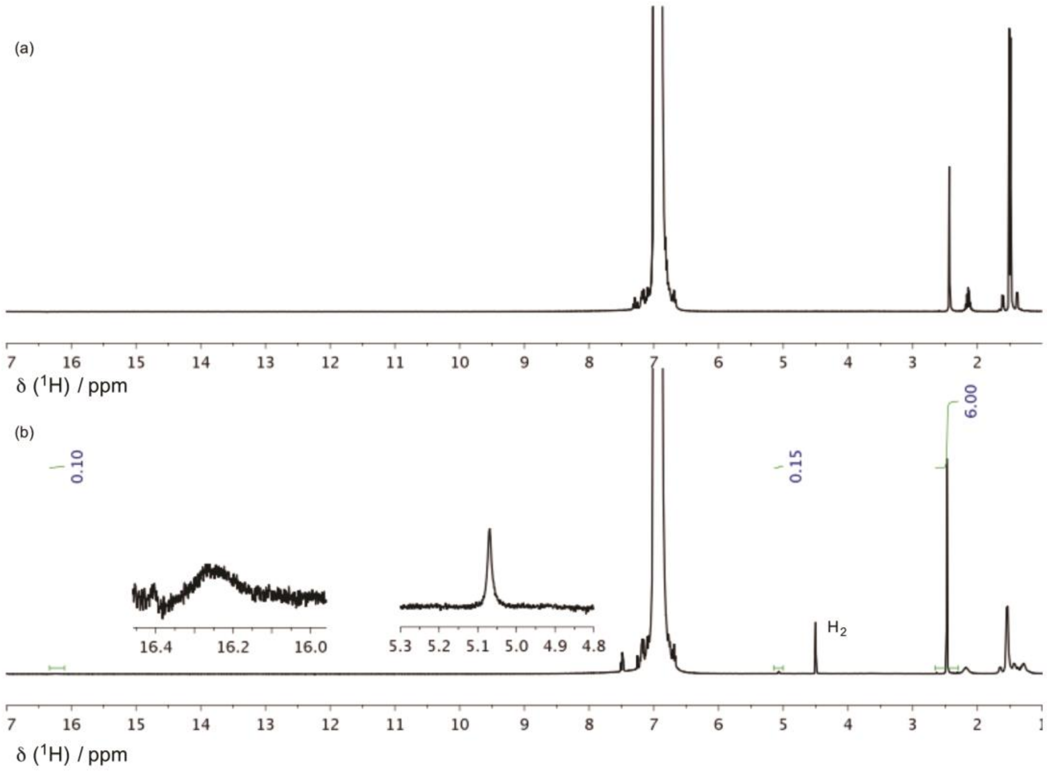

Figure S8 – ^{1}H NMR spectra of [**1**]OTf / lutidine in 1,2-difluorobenzene before (a) and after (b) admission and activation of H_2 (10 bar)

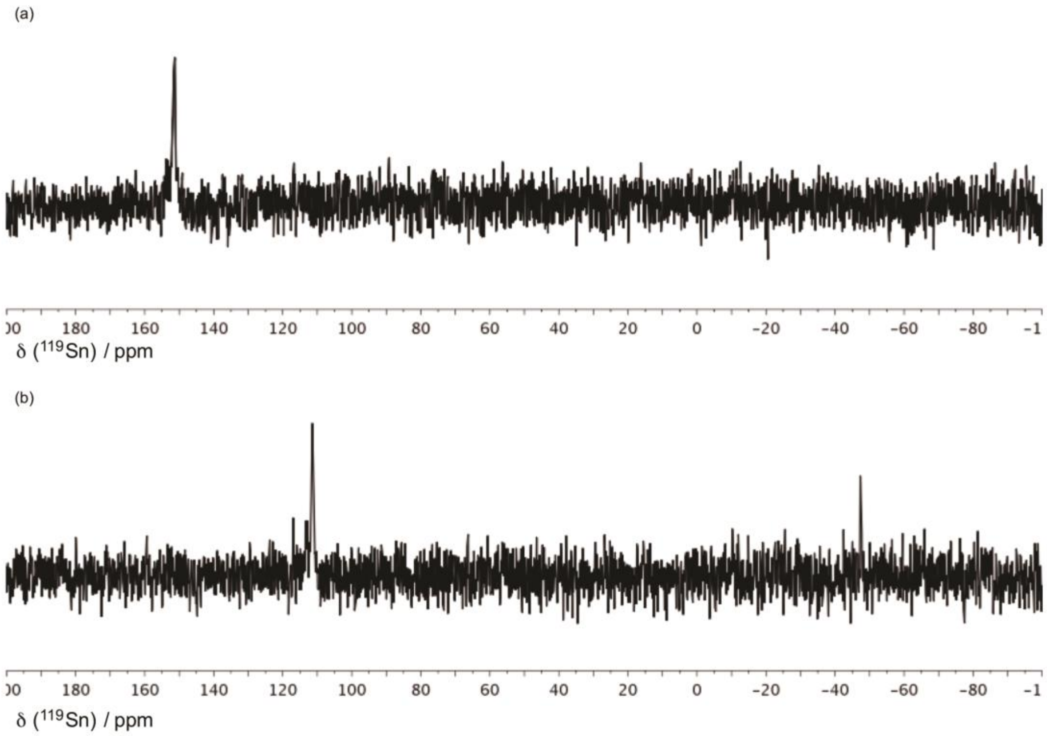

Figure S9 – ^{119}Sn{^{1}H} NMR spectra of [**1**]OTf / lutidine in 1,2-difluorobenzene before (a) and after (b) admission and activation of H_2 (10 bar)

4.4. H_2 activation with collidine

[**1**]OTf (15.9 mg, 0.04 mmol) and collidine (5.3 μL, 0.04 mmol) were dissolved in 1,2-difluorobenzene (0.7 mL) in a Wilmad high pressure NMR tube fitted with a PV-ANV PTFE valve. H_2 was admitted up to a pressure of 10 bar (at RT), and the sample was allowed to stand for 20 hours (with occasional agitation) before being re-analysed. Note that due to slow relaxation of the Sn*H* resonance (*ca.* 14 s, measured *in situ*), the final ^{1}H NMR spectrum was recorded using an extended delay of 100s.

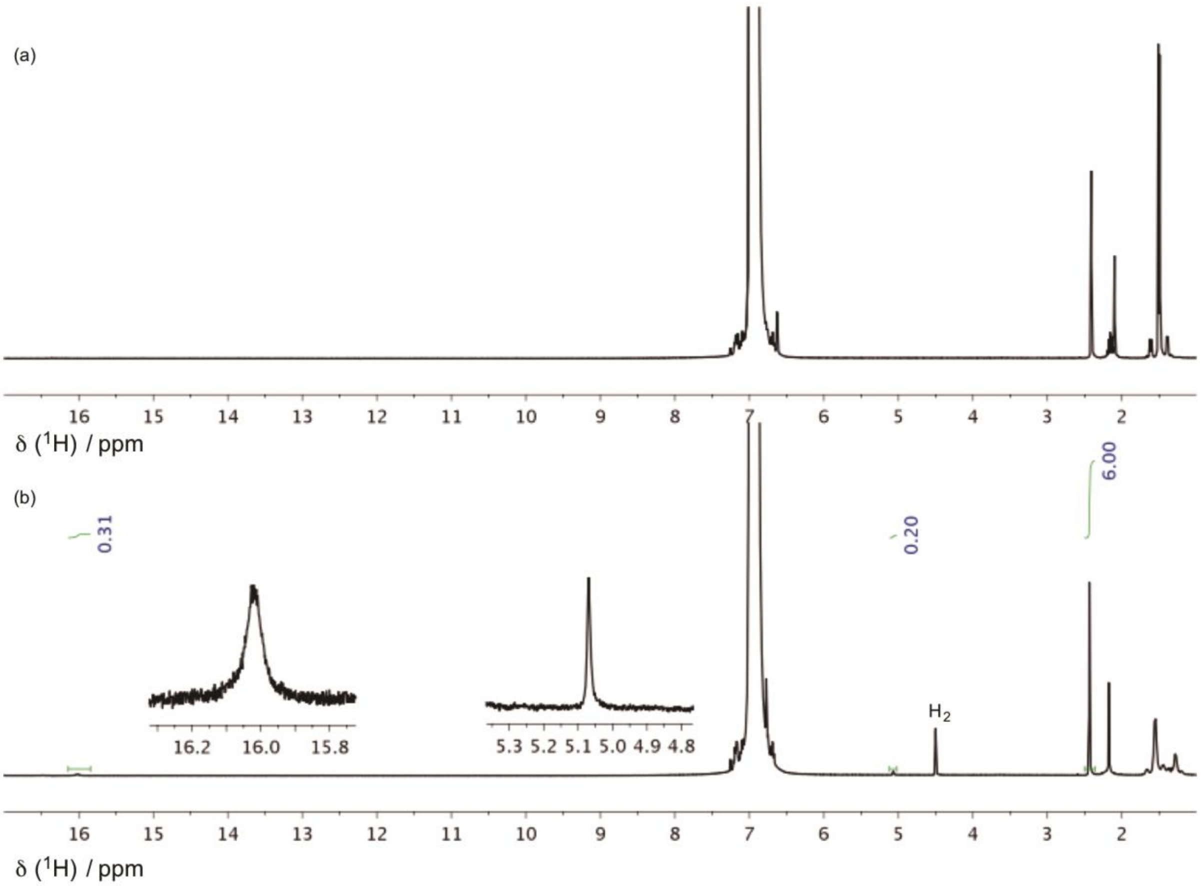

Figure S10 – ^{1}H NMR spectra of [**1**]OTf / collidine in 1,2-difluorobenzene before (a) and after (b) admission and activation of H_2 (10 bar)

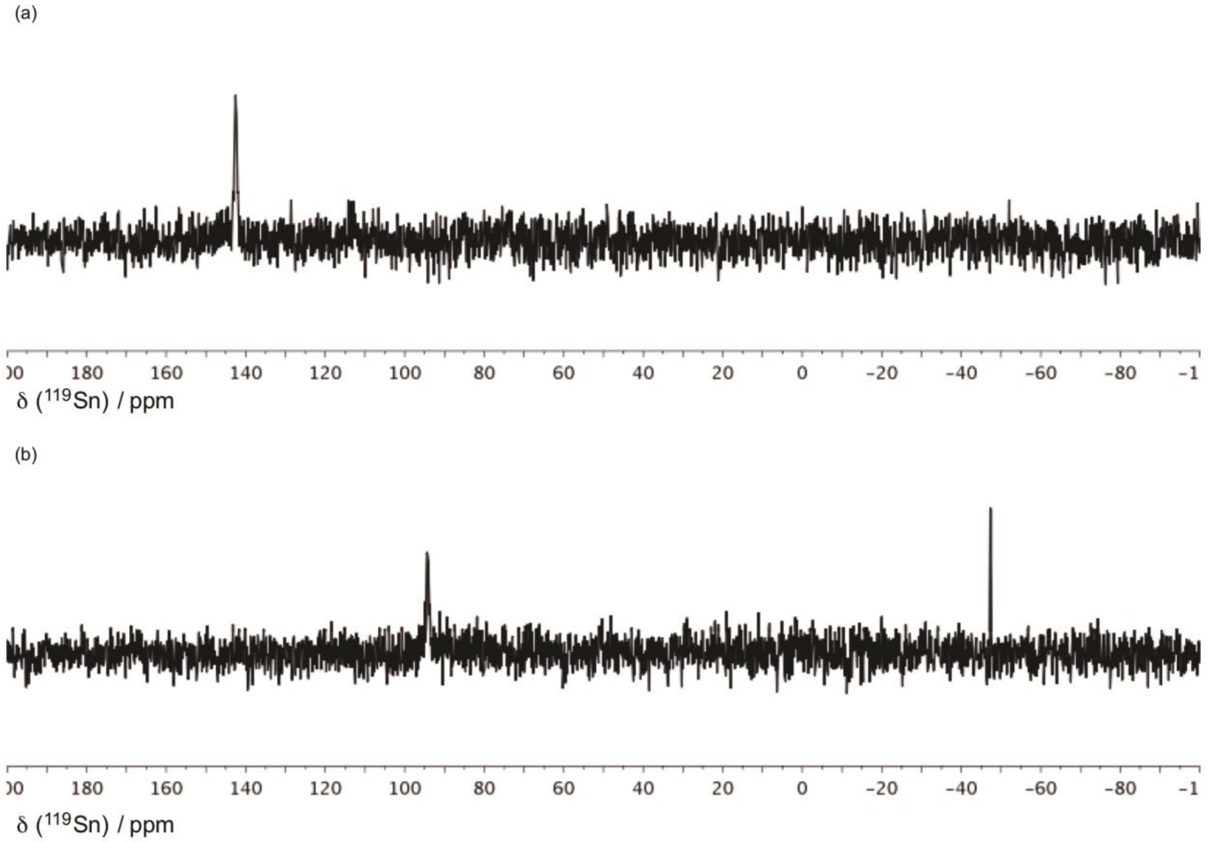

Figure S11 – ^{119}Sn{^{1}H} NMR spectra of [**1**]OTf / collidine in 1,2-difluorobenzene before (a) and after (b) admission and activation of H_2 (10 bar)

5. Typical procedure for hydrogenation of imines 2 catalysed by [1]OTf

A solution of imine (0.2 mmol) and, if necessary, collidine (2.6 μL, 0.02 mmol) in 1,2-dichlorobenzene (0.7 mL) was added to *i*Pr_3SnOTf ([**1**]OTf, 7.9 mg, 0.02 mmol) in a Wilmad high pressure NMR tube fitted with a PV-ANV PTFE valve. H_2 was admitted up to a pressure of 10 bar (at RT). The reaction mixture was heated in an oil bath as indicated in Table 1.

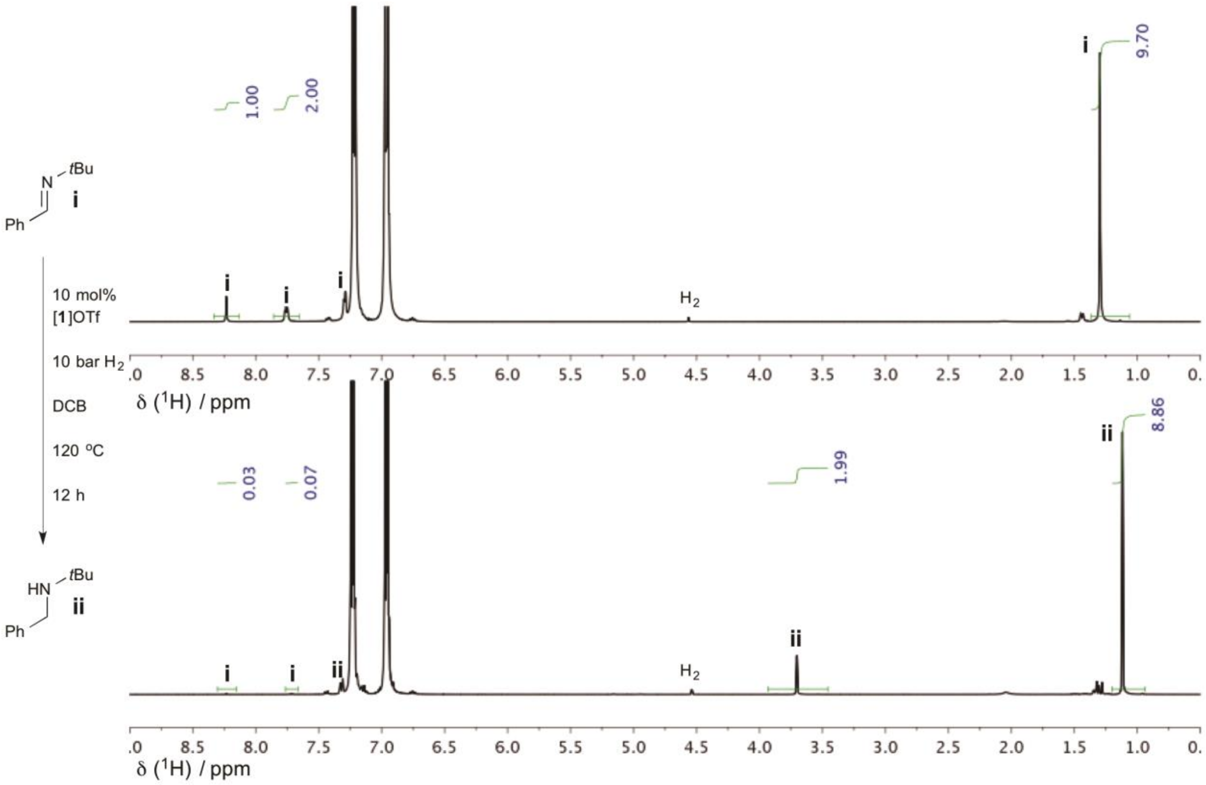

Figure S12 – ^{1}H NMR spectra for the hydrogenation of **2a** (final spectrum taken at 70 ºC to ensure homogeneity)

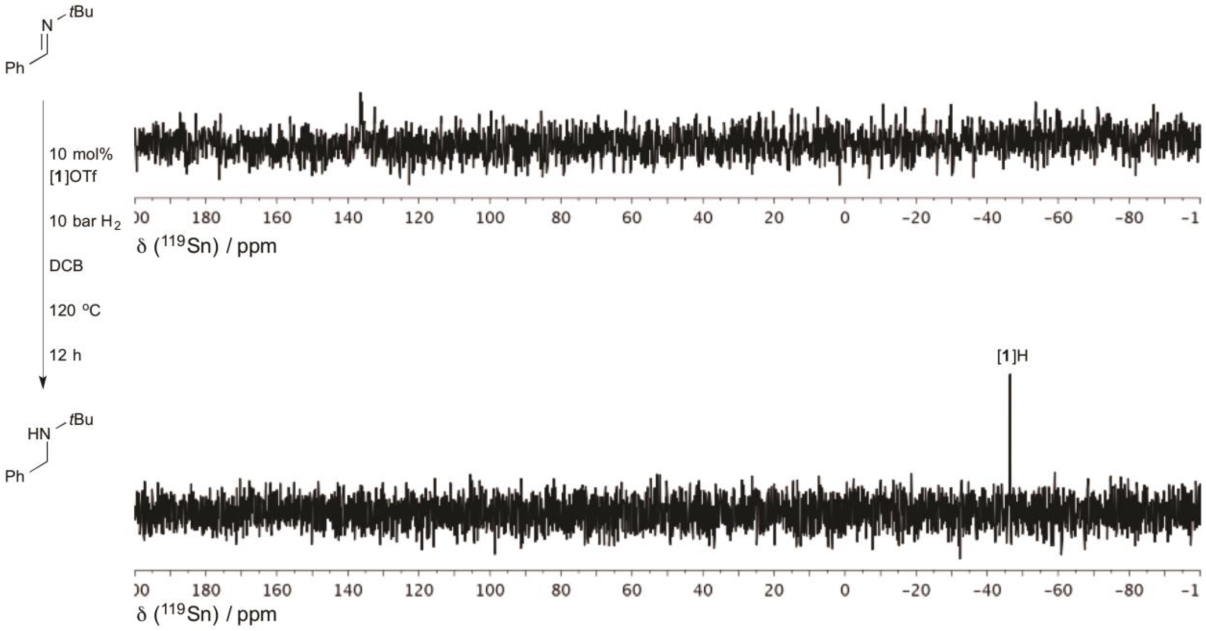

Figure S13 – ^{119}Sn{^{1}H} NMR spectra for the hydrogenation of **2a** (final spectrum taken at 70 ºC to ensure homogeneity)

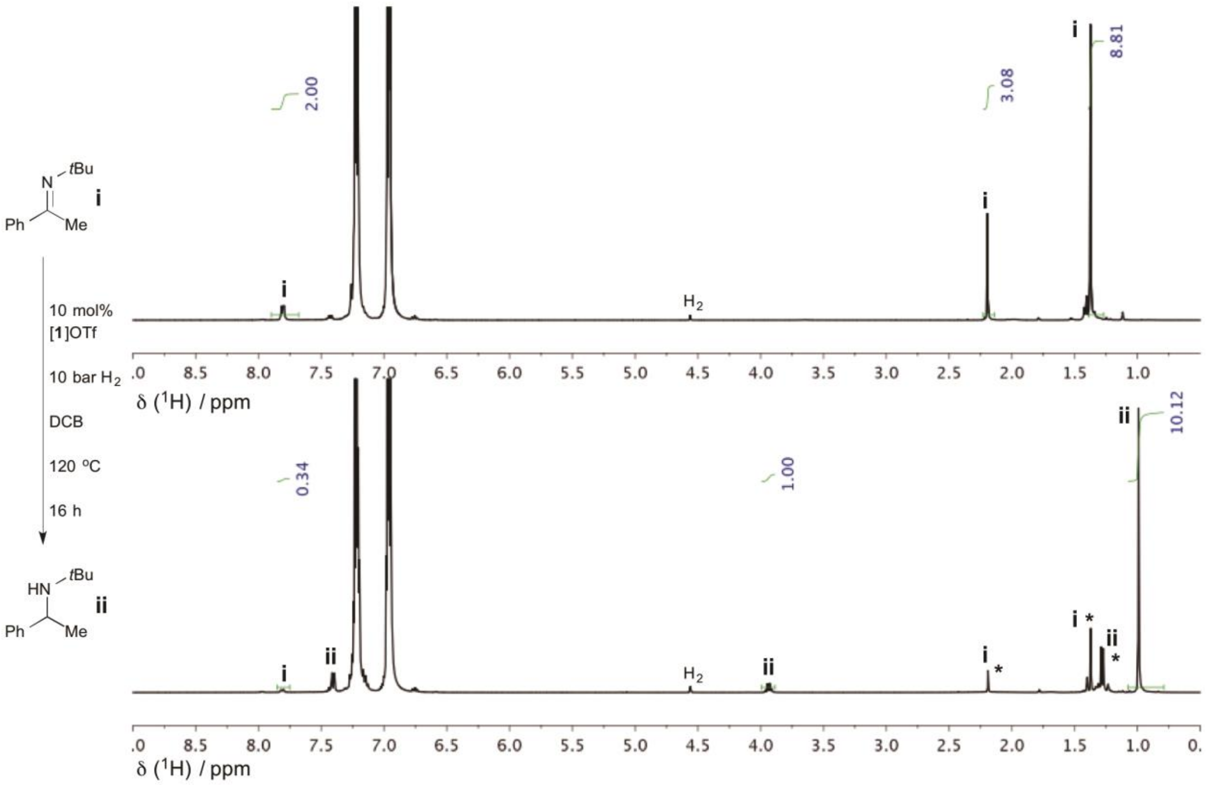

Figure S14 – ^{1}H NMR spectra for the hydrogenation of **2b** (note that the peaks marked * overlap with broad N*H* or catalyst-based resonances)

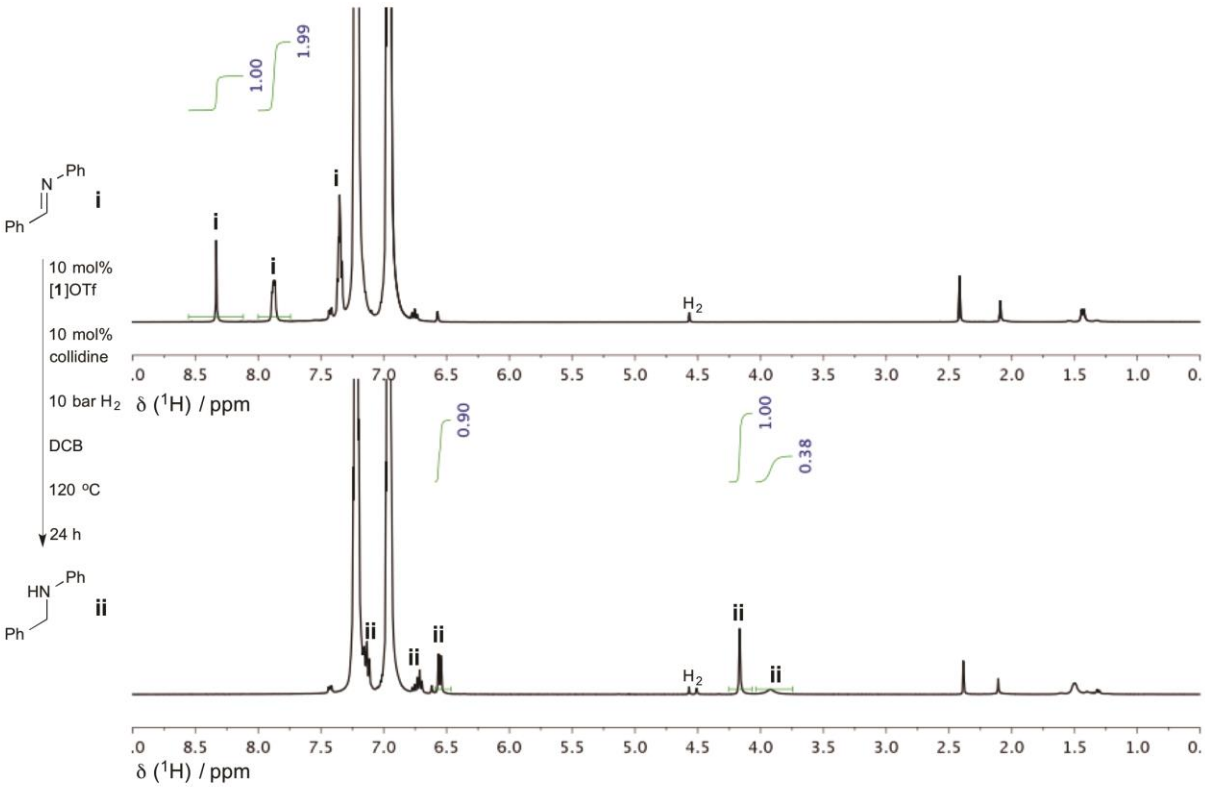

Figure S15 – ^{1}H NMR spectra for the hydrogenation of **2c**

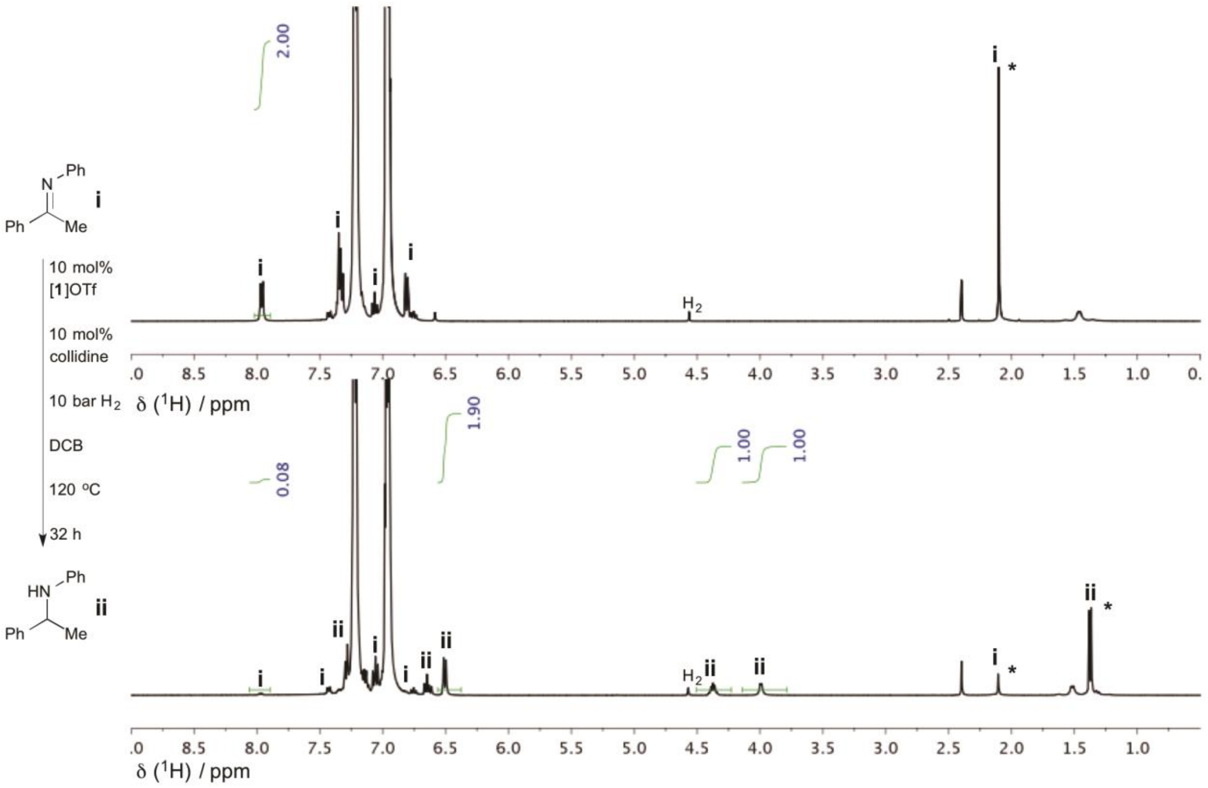

Figure S16 – ^{1}H NMR spectra for the hydrogenation of **2d** (note that the peaks marked * overlap with catalyst-based resonances)

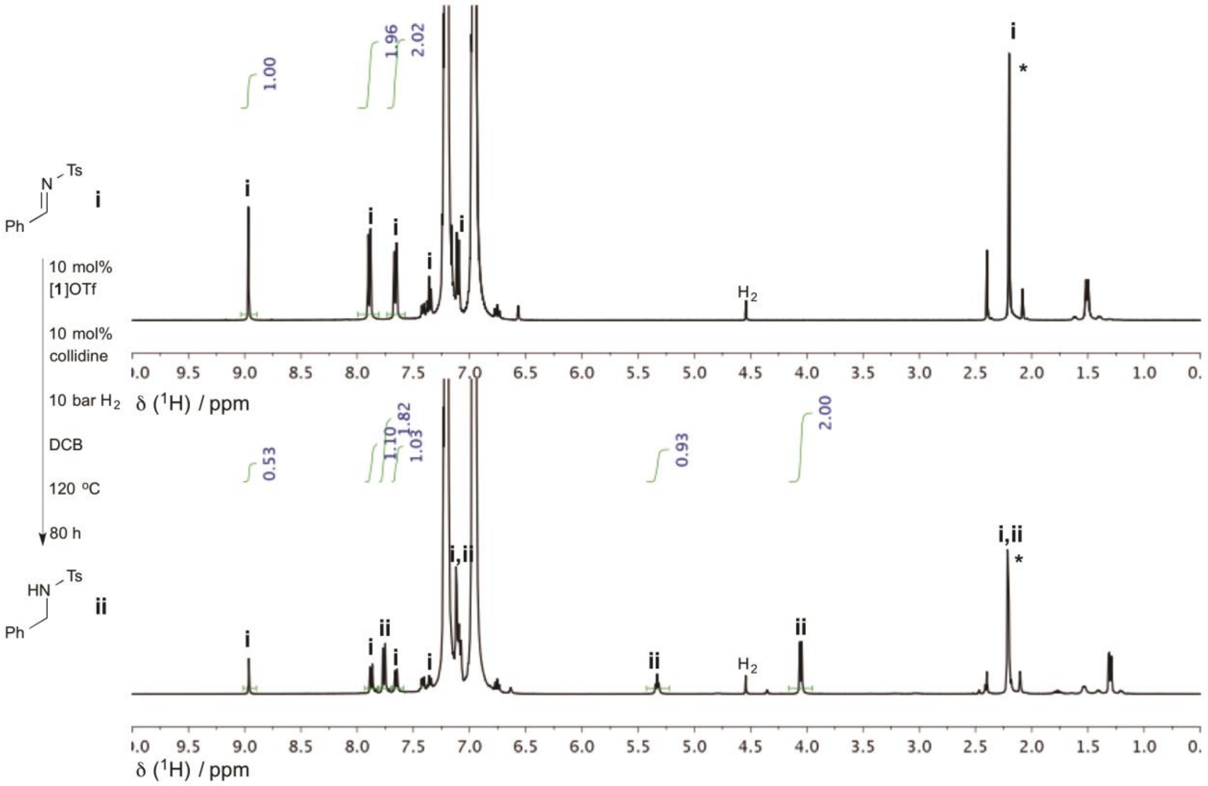

Figure S17 – ^{1}H NMR spectra for the hydrogenation of **2e** (note that the peaks marked * overlap with catalyst-based resonances)

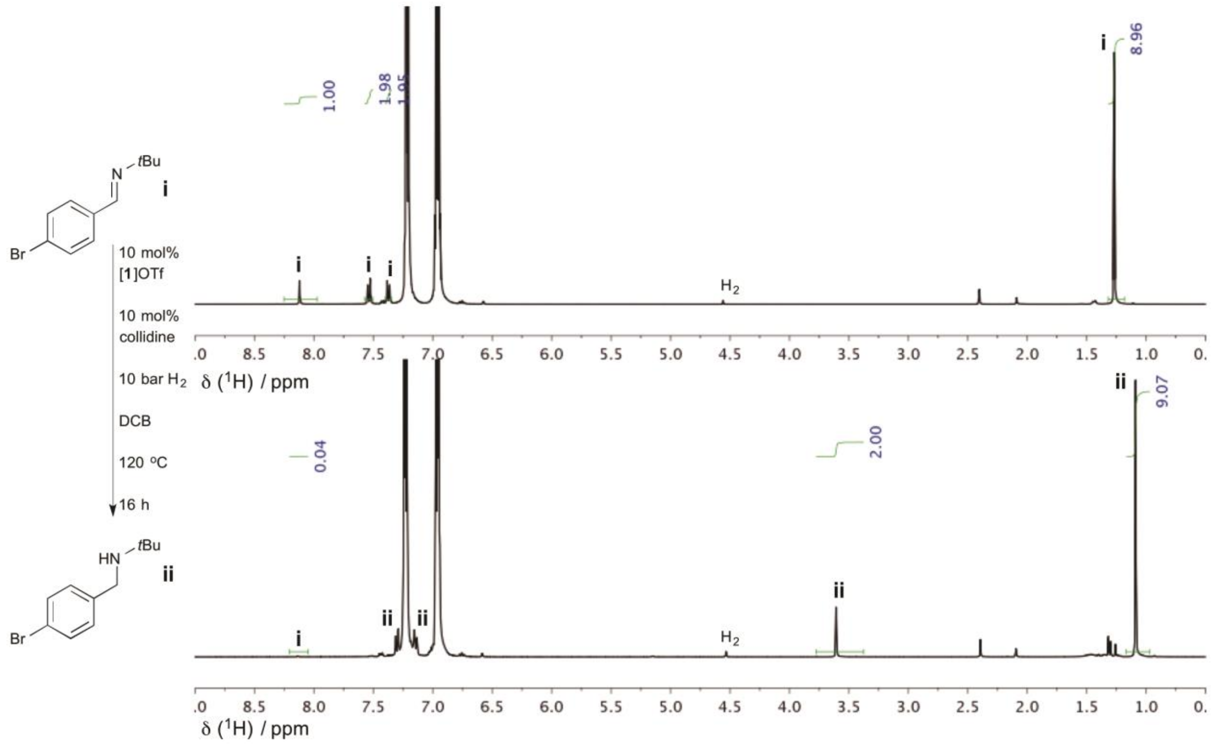

Figure S18 – ^{1}H NMR spectra for the hydrogenation of **2f** (final spectrum taken at 70 ºC to ensure homogeneity)

6. Proposed mechanism for hydrogenation of imines 2 by [1]OTf

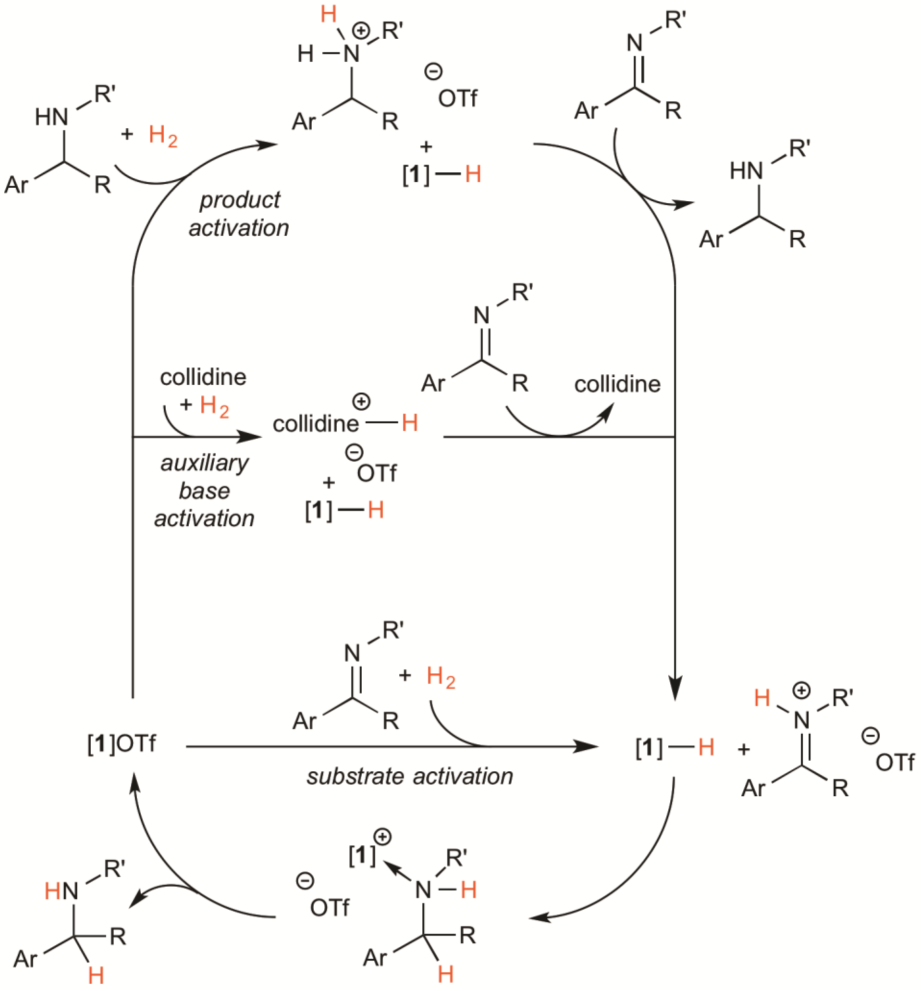

Figure S19 – General mechanism proposed for [**1**]OTf-catalysed imine hydrogenation (substrate and product activation pathways favoured for more basic imines; auxiliary base activation pathway favoured for less basic imines)

6.1. Stoichiometric addition of ^{1}H to 2a

To a solution of [**1**]H (5.0 mg, 0.02 mmol) in 1,2-dichlorobenzene (0.7 mL) in an NMR tube fitted with a J. Young's valve was added imine **2a** (3.6 μL, 0.02 mmol). No reaction was observed by ^{1}H or ^{119}Sn{^{1}H} NMR spectroscopy, even after heating to 120 ºC for 17 h.

6.2. Stoichiometric addition of ^{1}H to 2a·HOTf

To a solution of HOTf (3.0 mg, 0.02 mmol) in 1,2-dichlorobenzene (0.35 mL) in an NMR tube fitted with a J. Young's valve was added imine **2a** (3.6 μL, 0.02 mmol). The resulting mixture was agitated by hand, resulting in immediate formation of a colourless precipitate, and left to stand at RT for 24 h.

To this was added [**1**]H (5.0 mg, 0.02 mmol) in 1,2-dichlorobenzene (0.35mL). The mixture was again agitated by hand, becoming fully homogeneous within *ca*. 1 min.

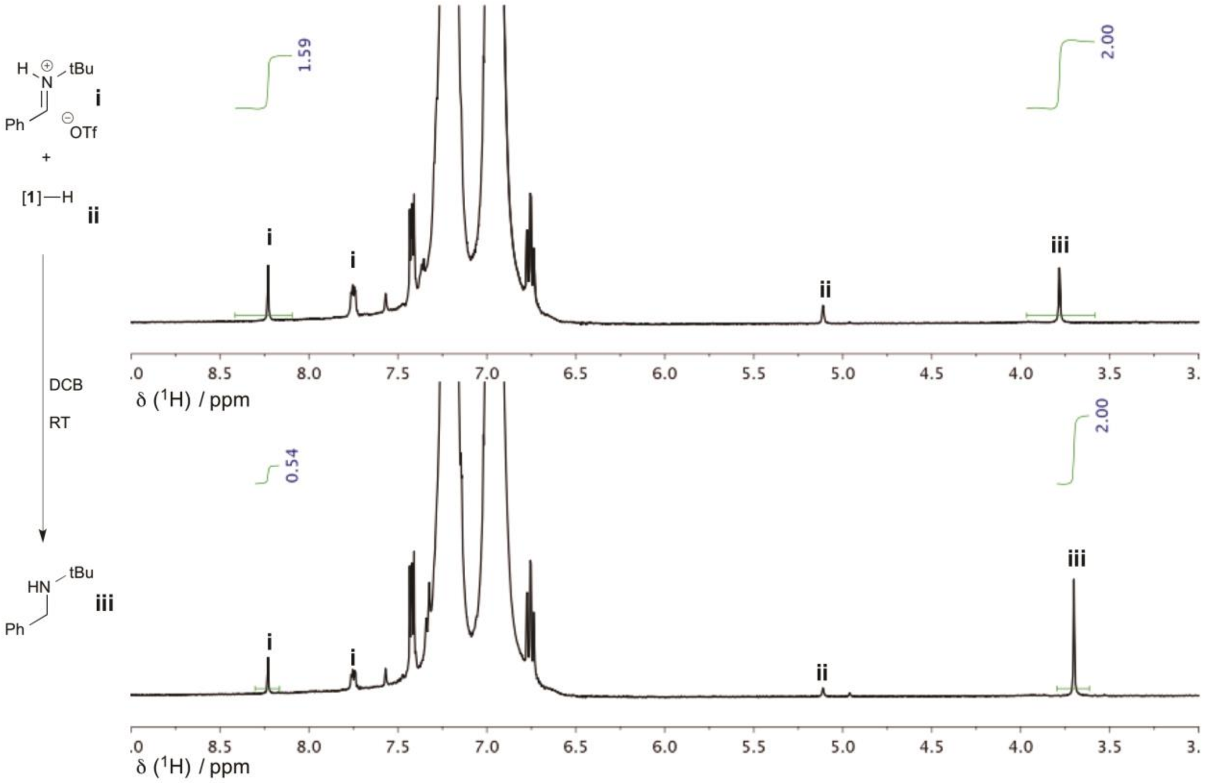

Figure S20 – ^{1}H NMR spectra for the reaction of **2a**·HOTf with [**1**]H after (a) a few minutes, and (b) 17 h (^{119}Sn{^{1}H} NMR spectroscopy showed only the expected [**1**]H resonance at -49 ppm)

6.3. Stoichiometric addition of ^{1}H to 2a / [1]OTf

To a suspension of [**1**]OTf (7.9 mg, 0.02 mmol) and imine **2a** (3.6 μL, 0.02 mmol) in 1,2-dichlorobenzene (0.7 mL) in an NMR tube fitted with a J. Young's valve was added [**1**]H (5.0 mg, 0.02 mmol). No significant change was observed by ^{1}H or ^{119}Sn{^{1}H} NMR spectroscopy, even after heating to 120 ºC for 70 h.

7. Typical procedure for hydrogenation of aldehydes and ketones 4 catalysed by [1]OTf

A solution of substrate **4** (0.4 mmol) and base (0.04 mmol) in 1,2-dichlorobenzene (0.7 mL) was added to *i*Pr_3SnOTf ([**1**]OTf, 15.9 mg, 0.04 mmol) in a Wilmad high pressure NMR tube fitted with a PV-ANV PTFE valve. For **4b** and **4c** a drop of $SiMe_4$ was also added to act as an internal integration standard. H_2 was admitted up to a pressure of 10 bar (at RT). The reaction mixture was heated in an Al bead bath as indicated in Table 2. In certain cases the reaction was periodically removed from the heating bath and repressurised to 10 bar with H_2.

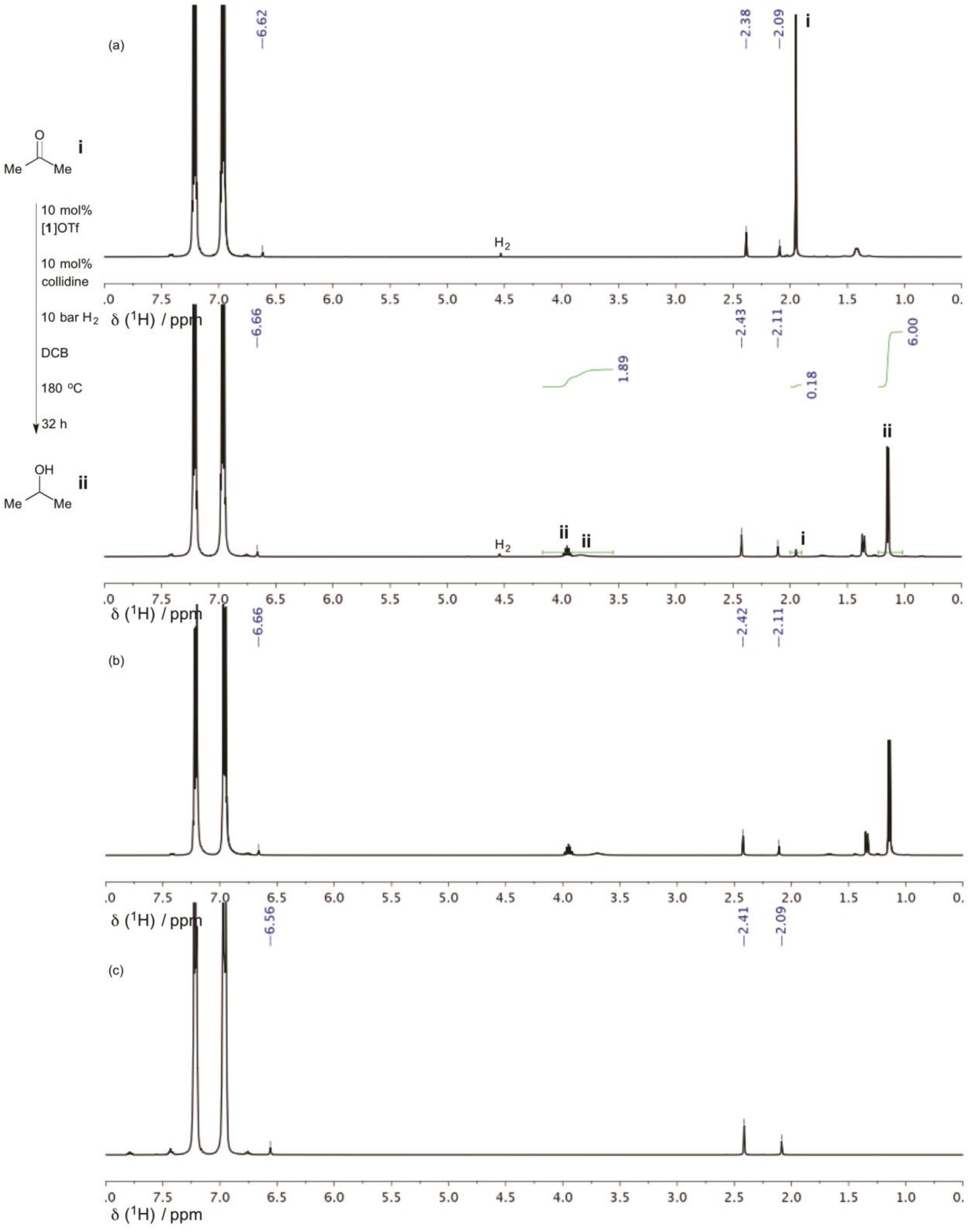

Figure S21 – ^{1}H NMR spectra (a) for the hydrogenation of **4a**, emphasising the downfield shift of the collidine-derived resonances; (b) of an independently prepared sample of **5a** (10 eq.), [**1**]OTf and collidine at the same concentration in 1,2-dichlorobenzene; (c) of a sample of collidine alone.

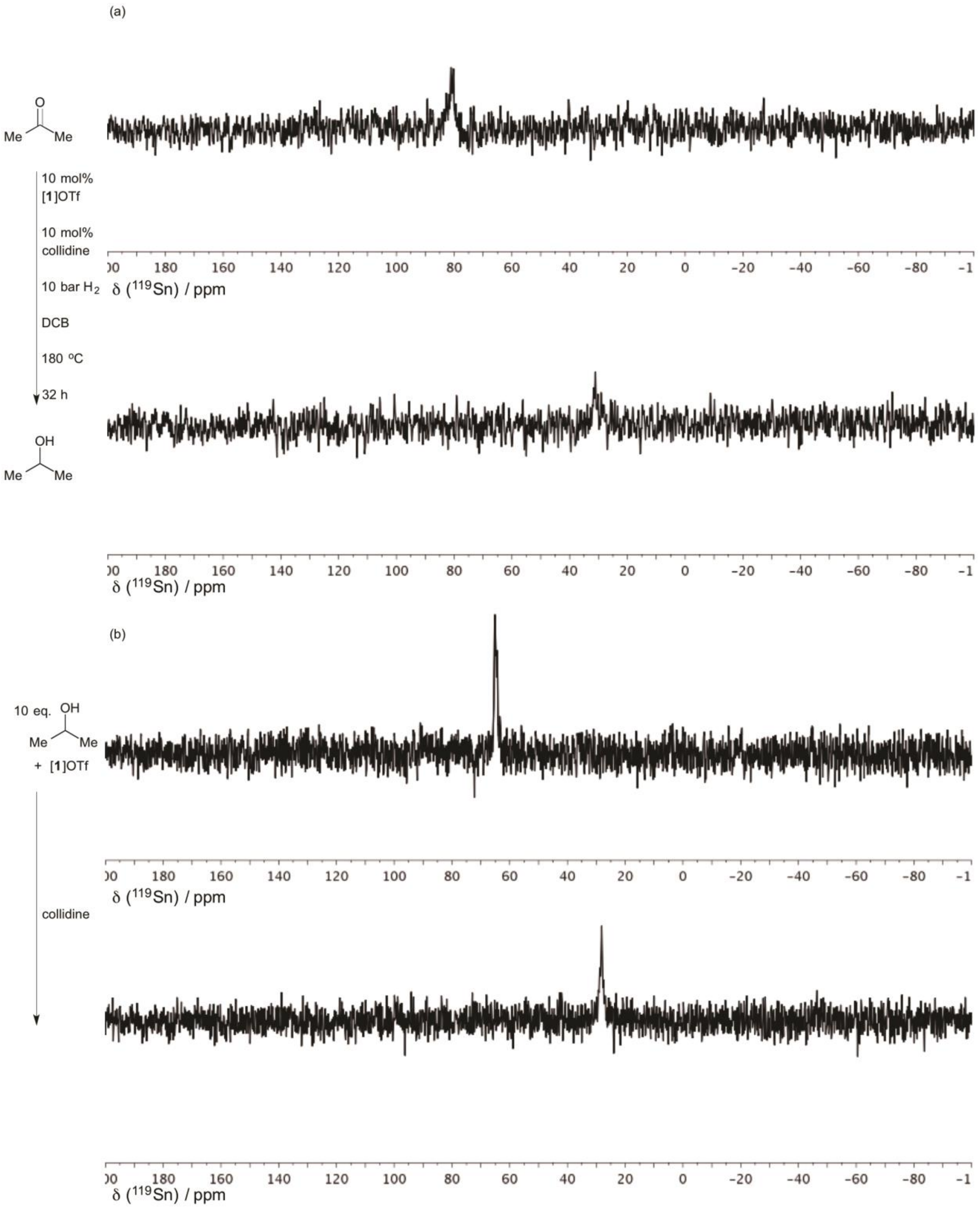

Figure S22 – ^{119}Sn{^{1}H} NMR spectra (a) for the hydrogenation of **4a**; (b) of an independently prepared sample of **5a** (10 eq.) and [**1**]OTf at the same concentration in 1,2-dichlorobenzene, before and after addition of collidine.

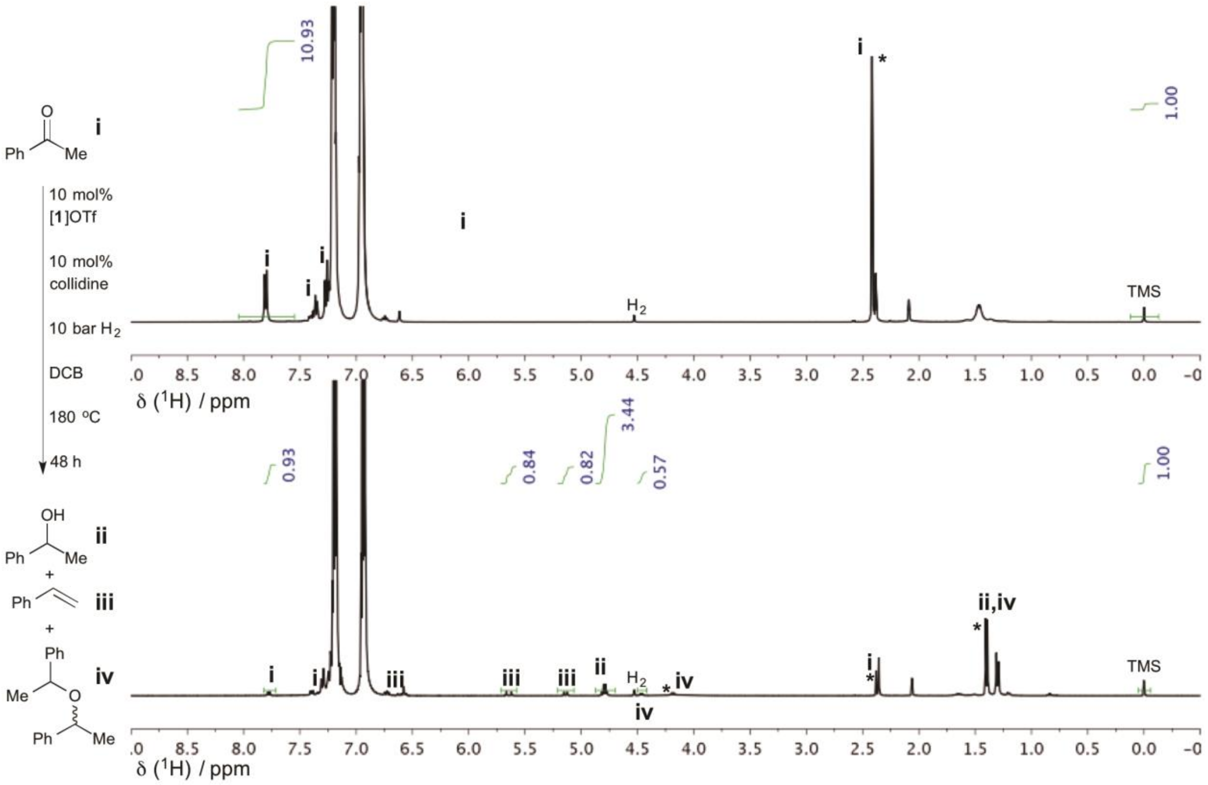

Figure S23 – ^{1}H NMR spectra for the hydrogenation of **4b**. Note that the peaks marked * overlap with O*H* or catalyst-based resonances. The molar ratio of **ii**:**iii**:**iv** (**5b**:**6**:**7**) is 74:18:8 (obscured resonances prevent direct integration of the total intensity for **iv**/**7**, so the ratio is calculated assuming **iv**/**7** accounts for all of the remaining intensity lost relative to the TMS internal standard).

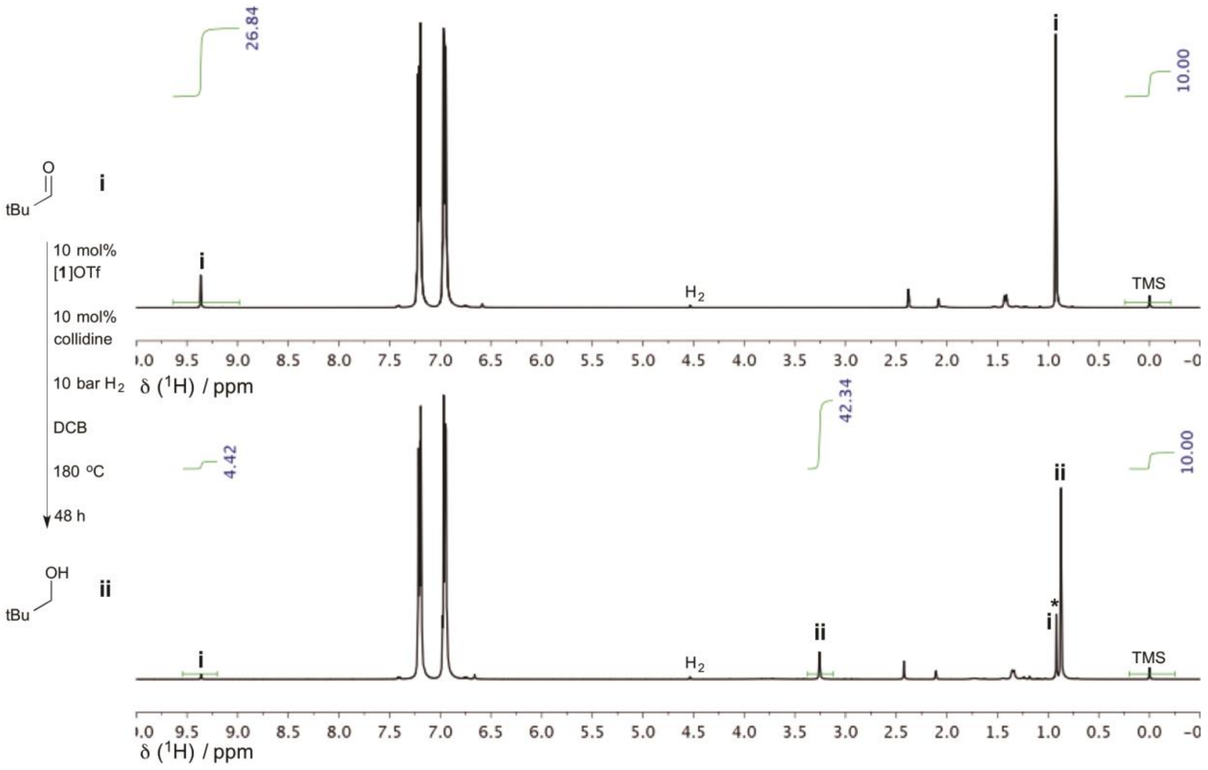

Figure S24 – ^{1}H NMR spectra for the hydrogenation of **4c** (note that the *t*Bu peaks marked * overlap)

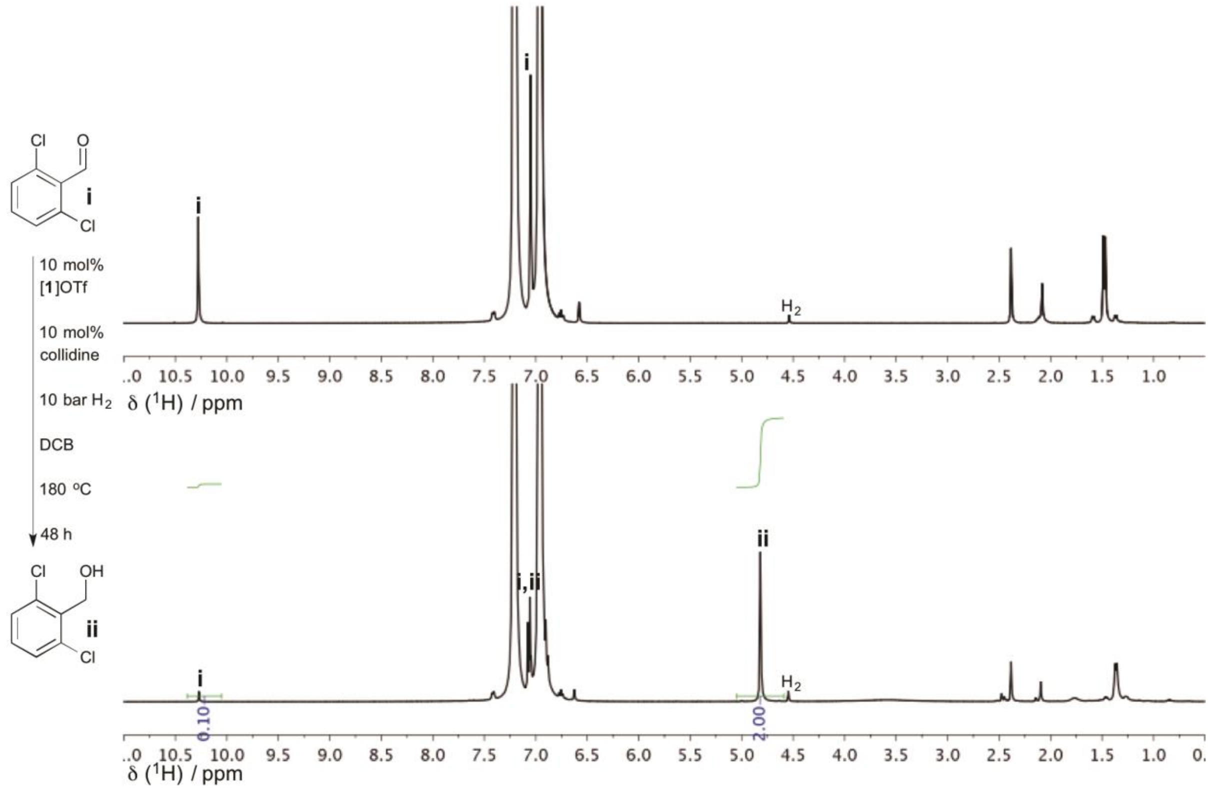

Figure S25 – ^{1}H NMR spectra for the hydrogenation of **4d**

8. Mechanistic investigations into the hydrogenation of 4a

8.1. Hydrogenation of 4a catalysed by [1]OTf / [1]O*i*Pr

To a solution of acetone (32 μL, 0.44 mmol) and [**1**]OTf (15.9 mg, 0.04 mmol) in 1,2-dichlorobenzene (0.7 mL) in a Wilmad high pressure NMR tube fitted with a PV-ANV PTFE valve, was added [**1**]H (10.0 mg, 0.04 mmol). H_2 was admitted up to a pressure of 10 bar (at RT). Initial NMR spectroscopic analysis indicated complete consumption of [**1**]H to form [**1**]O*i*Pr. The reaction mixture was heated in an Al bead bath to 180 ºC for 16 h.

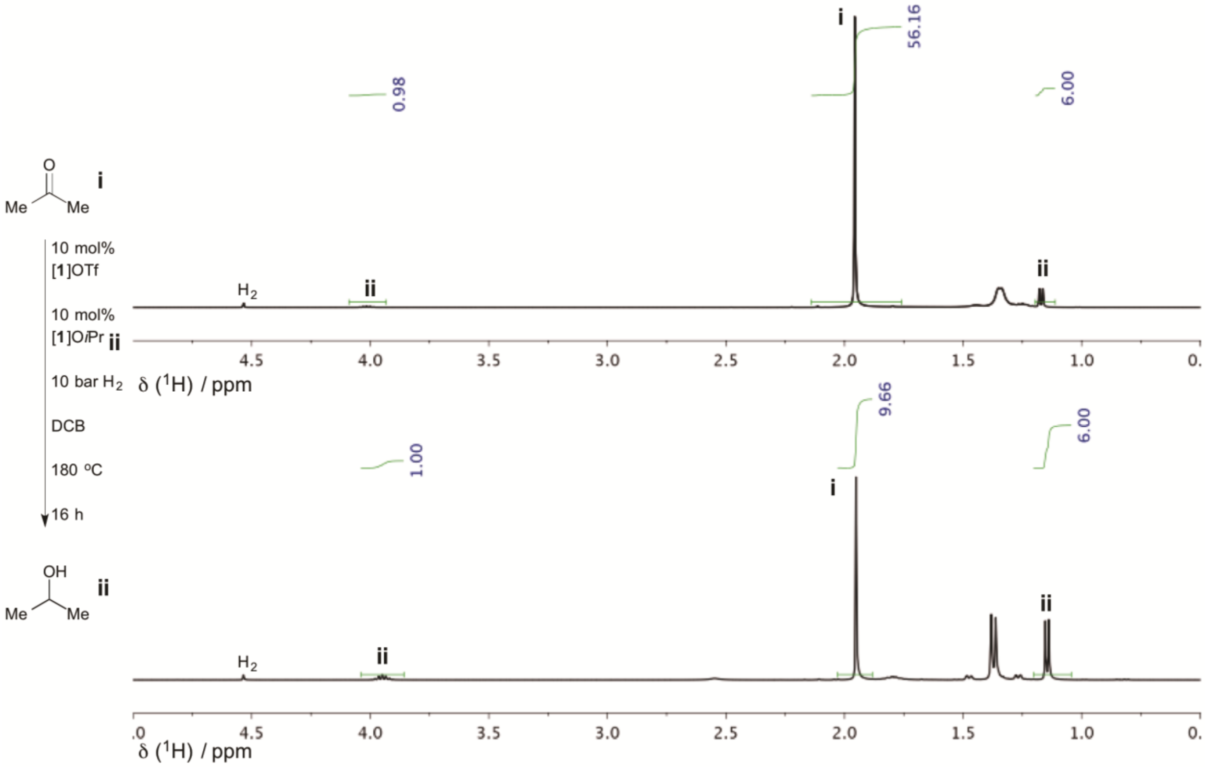

Figure S26 – ^{1}H NMR spectra for the hydrogenation of **4a** by [**1**]OTf / [**1**]O*i*Pr

8.2. Stoichiometric addition of ^{1}H to 4a

To a solution of [**1**]H (5.0 mg, 0.02 mmol) in 1,2-dichlorobenzene (0.7 mL) in an NMR tube fitted with a J. Young's valve was added acetone, **4a** (1.5 µL, 0.02 mmol). No reaction was observed by ^{1}H or ^{119}Sn{^{1}H} NMR spectroscopy, even after heating to 120 ºC for 17 h.

8.3. Stoichiometric addition of ^{1}H to 4a / collidine·HOTf

To a solution of HOTf (3.0 mg, 0.02 mmol) and collidine (2.6 µL, 0.02 mmol) in 1,2-dichlorobenzene (0.7 mL) in an NMR tube fitted with a J. Young's valve was added acetone, **4a** (1.5 µL, 0.02 mmol), followed by [**1**]H (5.0 mg, 0.02 mmol). The resulting mixture was left to stand at RT for 24 h, then heated to 120 ºC for 17h.

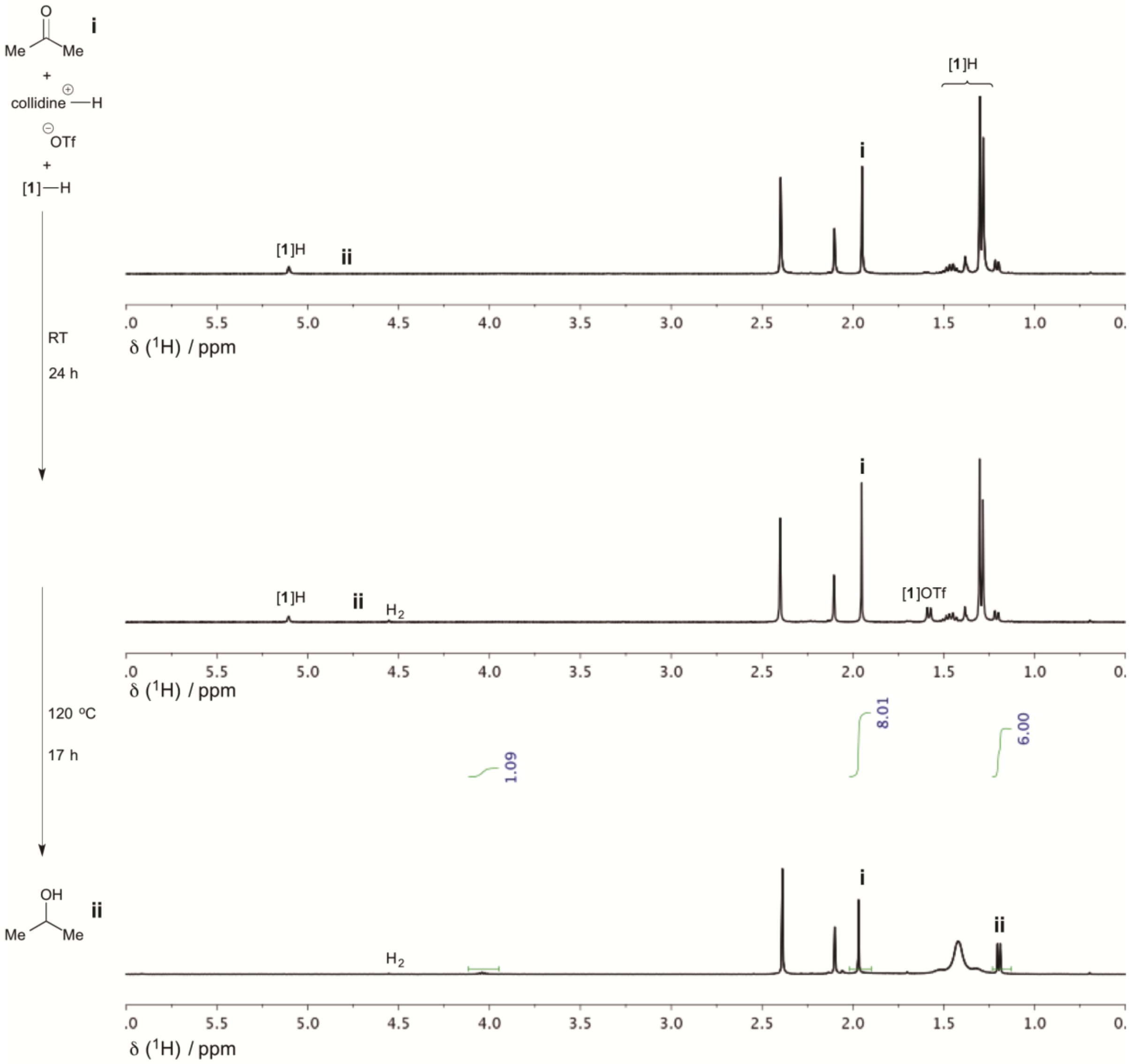

Figure S27 – ^{1}H NMR spectra for the reaction of [**1**]H with **4a** / collidine·HOTf

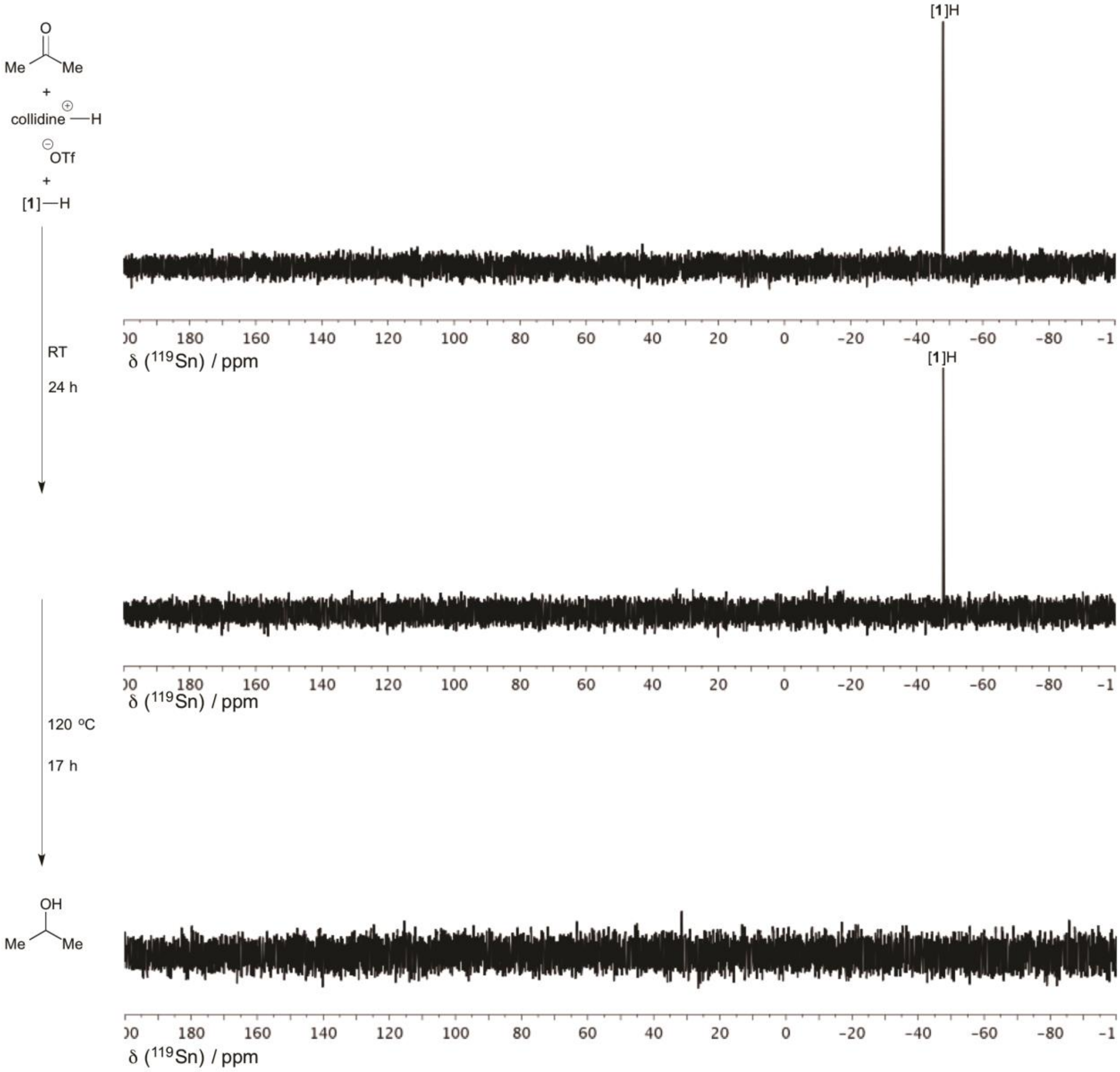

Figure S28 – ^{119}Sn{^{1}H} NMR spectra for the reaction of [**1**]H with **4a** / collidine·HOTf

8.4. Stoichiometric addition of ^{1}H to 4a / [1]OTf

To a suspension of [**1**]OTf (7.9 mg, 0.02 mmol) and acetone, **4a** (1.5 μL, 0.02 mmol), in 1,2-dichlorobenzene (0.7 mL) in an NMR tube fitted with a J. Young's valve was added [**1**]H (5.5 mg, 0.022 mmol). NMR spectroscopic analysis showed immediate reduction. Further reduction was significantly slower, presumably due to the poor solubility of [**1**]OTf under these conditions, but still proceeded to completion overnight at RT.

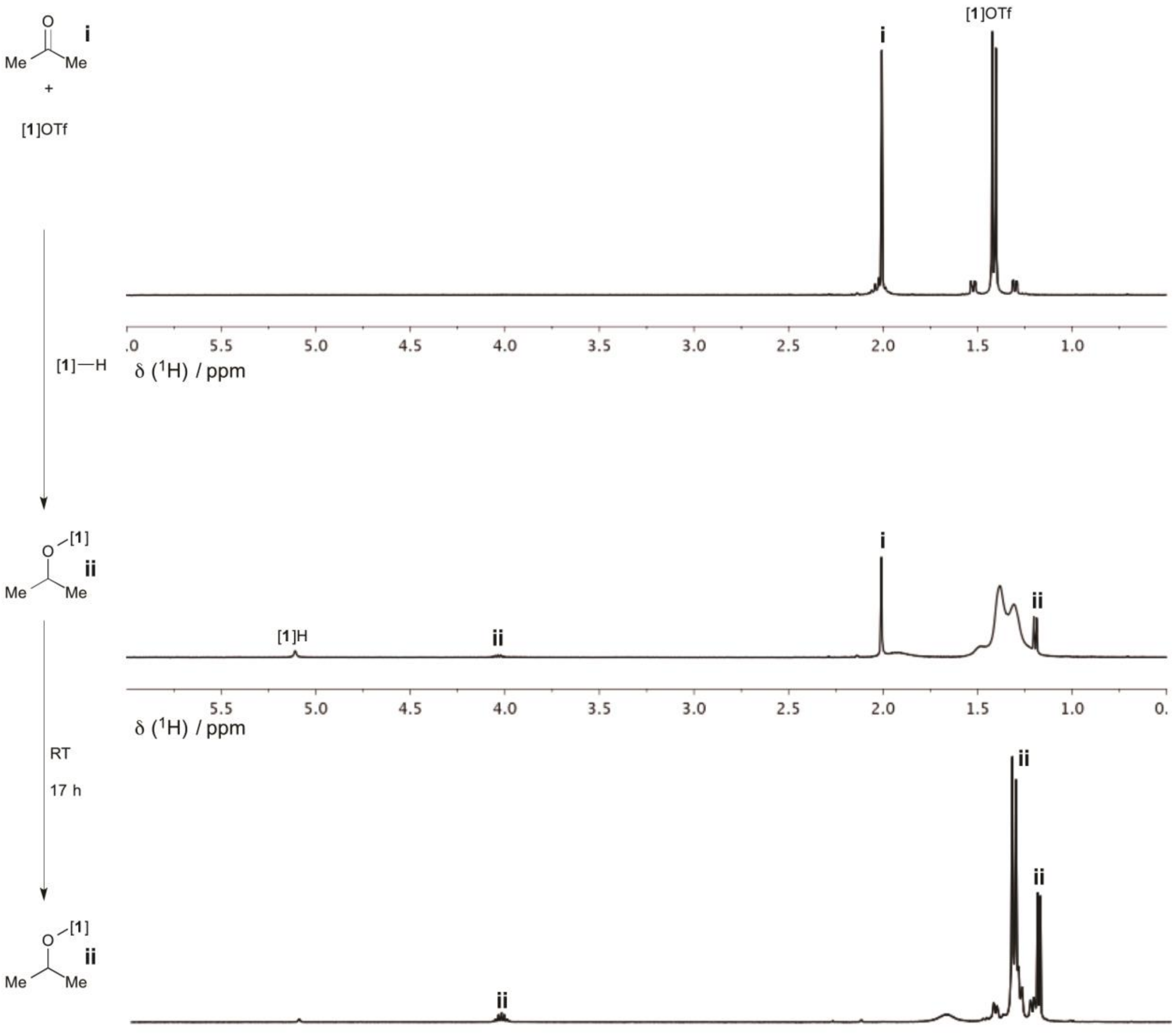

Figure S29 – 1H NMR spectra for the reaction of [**1**]H with **4a** / [**1**]OTf

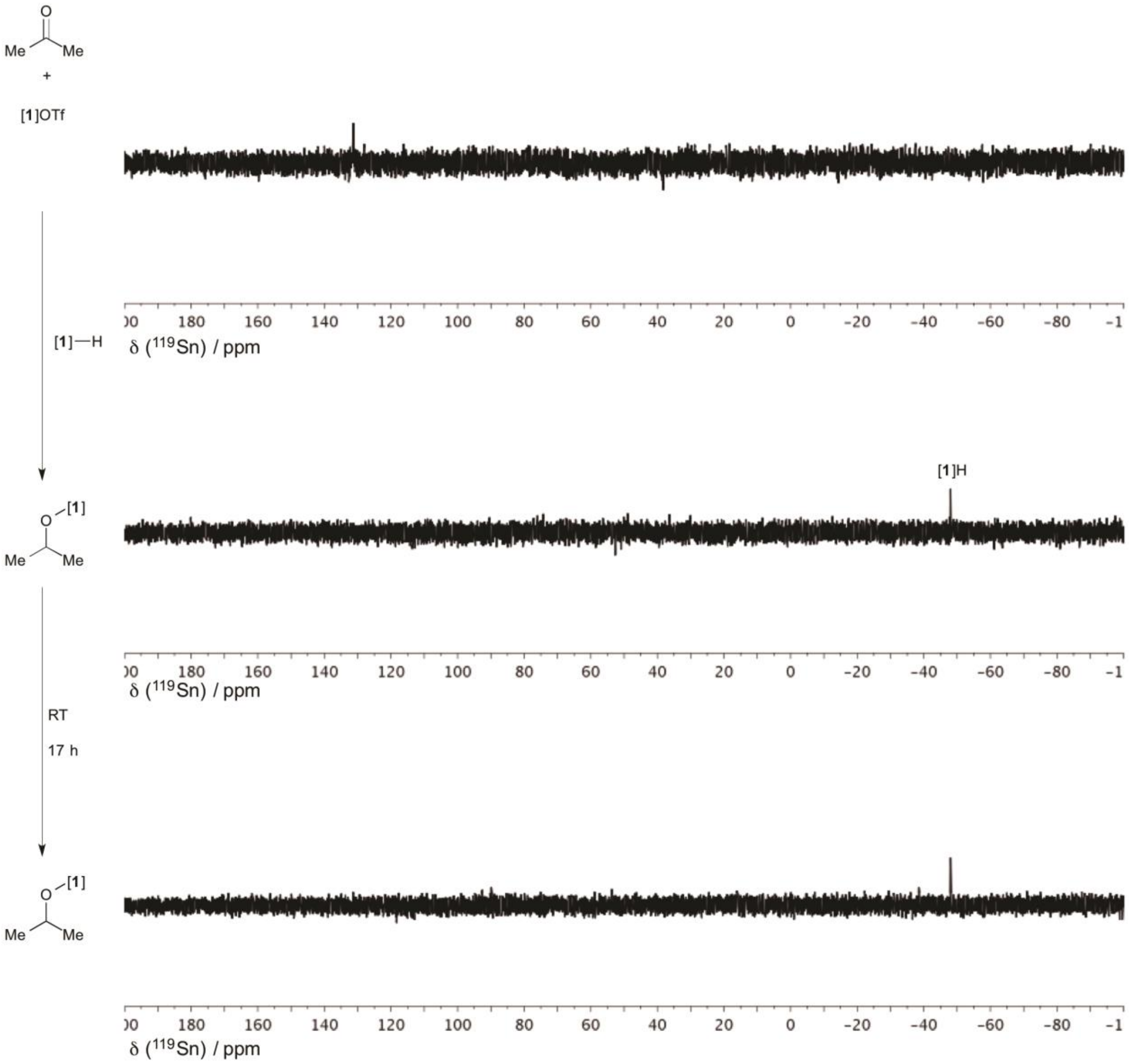

Figure S30 – $^{119}Sn\{^1H\}$ NMR spectra for the reaction of [**1**]H with **4a** / [**1**]OTf

8.5. A note on the mechanism of carbonyl hydrogenation, and the effect of base strength

Although prior analysis has shown that combinations of [**1**]OTf and the bases employed for catalytic carbonyl hydrogenation are capable of activating H_2 under the reaction pressures to a sufficient extent for the activation products to be observed by NMR (see section 4, above), no [**1**]H was actually observed by ^1H or $^{119}Sn\{^1H\}$ NMR spectroscopy when analysing any of these reaction mixtures at partial conversion. Instead, $^{119}Sn\{^1H\}$ spectra (taken mostly at RT) suggest that the Sn catalyst is primarily sequestered in the form of the alkoxide [**1**]OR (e.g. [**1**]O*i*Pr for hydrogenation of **4a**, see figure S22). Given that hydride transfer from [**1**]H to the catalyst-activated substrate is expected to be very rapid (see above), this indicates that the rate of turnover is limited largely by the ability of the system to protonate off the alkoxide ligand, and free a small amount of the active Lewis acid catalyst to engage in further H_2 and substrate activation. The reduced rate of turnover with lutidine (versus collidine) can therefore be understood as the result of H_2 activation being less favourable with this

weaker base, while the reduced rate with DABCO can probably be attributed to less favourable protonation of the alkoxide.

9. 'Open bench' hydrogenation of 4a catalysed by [1]OTf

For this reaction, 1,2-dichlorobenzene, collidine and acetone (**4a**) were purchased from major suppliers (non-anhydrous grades) and used as supplied without further drying or other purification. H_2 was purchased from BOC (research grade) and used without further drying.

Inside the glovebox, *i*Pr_3SnOTf ([**1**]OTf, 15.9 mg, 0.04 mmol) was weighed out into a vial. The sample was removed from the glovebox and left to stand under air for a period of 1 week prior to use. Note that samples exposed to air in this manner appear to absorb 2 eq. of H_2O, based on ^{1}H NMR analysis, which may indicate formation of a 5-coordinate $[H_2O–\mathbf{1}–OH_2]^+$ cation, similar to previously-reported $[n\text{Bu}_3\text{Sn}(OH_2)_2]^+$.[8] To this sample was subsequently added 1,2-dichlorobenzene (0.7 mL), collidine (5.3 μL, 0.04 mmol) and **4a** (29 μL, 0.4 mmol) on the open bench. After shaking thoroughly the resulting homogeneous solution was transferred to a Wilmad high pressure NMR tube fitted with a PV-ANV PTFE valve (still under air), to which H_2 was then added up to a pressure of 10 bar (at RT). The reaction mixture was heated in an Al bead bath to 180 ºC for 16 h, before being repressurised with H_2 and heated for a further 16 h.

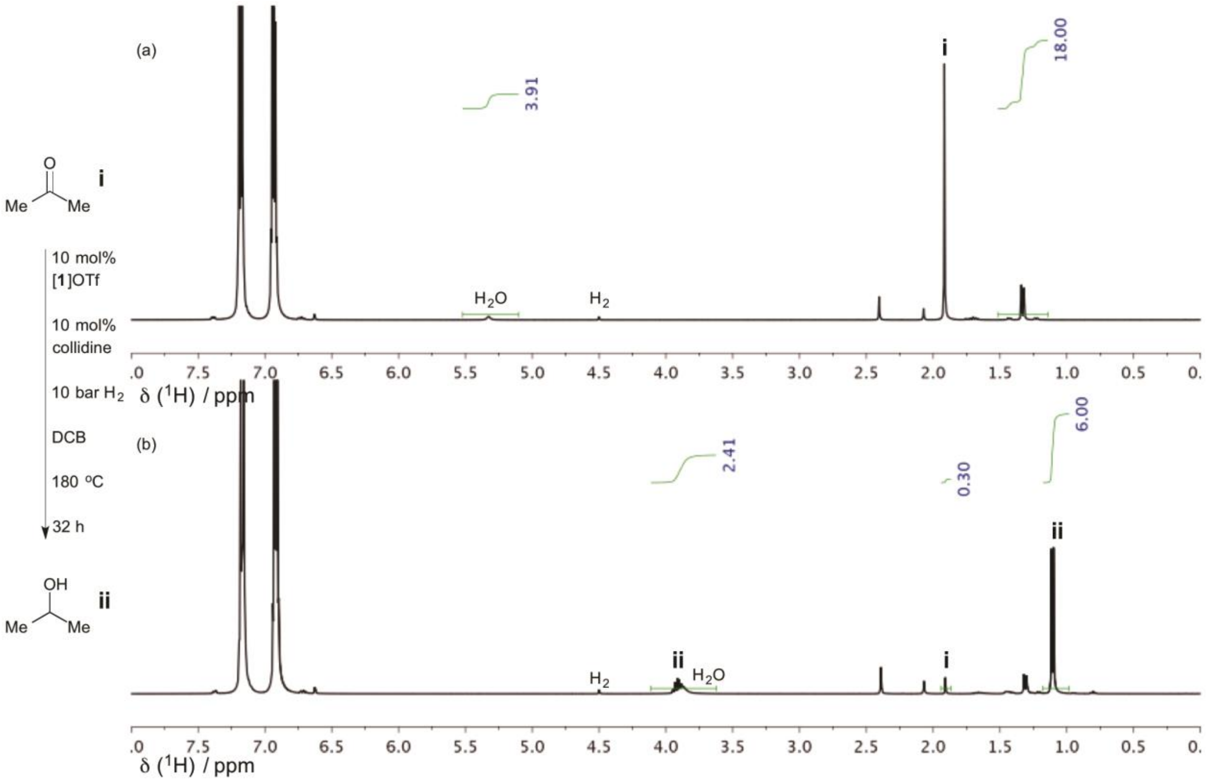

Figure S31 – ^{1}H NMR spectra for the hydrogenation of **4a** under 'open bench' conditions (note the resonance attributable to adventitious H_2O in the initial spectrum; integration suggests a 2:1 ratio of H_2O : [**1**]OTf)

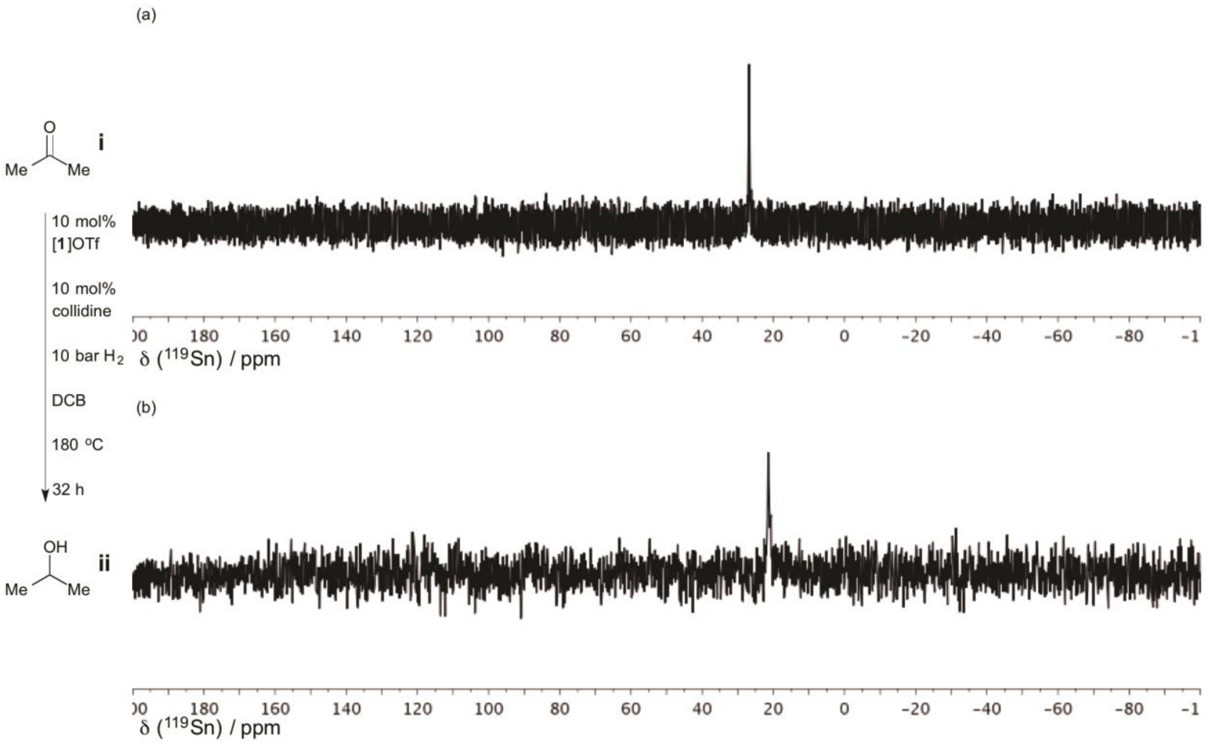

Figure S32 – ^{119}Sn{^{1}H} NMR spectra for the hydrogenation of **4a** under 'open bench' conditions

10. Hydrogenation of additional substrates catalysed by [1]OTf

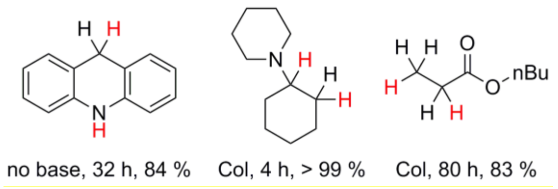

Figure S33 – Further examples of [**1**]OTf-catalysed hydrogenation. Conditions as described below. Highlighted H atoms derived from H_2. Conversions determined by ^{1}H NMR spectroscopic analysis.

10.1. Hydrogenation of acridine

A solution of acridine (35.8 mg, 0.2 mmol) in 1,2-dichlorobenzene (0.7 mL) was added to *i*Pr_3SnOTf ([**1**]OTf, 7.9 mg, 0.02 mmol) in a Wilmad high pressure NMR tube fitted with a PV-ANV PTFE valve. H_2 was admitted up to a pressure of 10 bar (at RT). The reaction mixture was heated in an oil bath to 120 ºC for 32 h. ^{1}H NMR spectroscopic analysis indicated 84 % conversion to acridane (final NMR spectrum recorded at 70 ºC to ensure homogeneity).

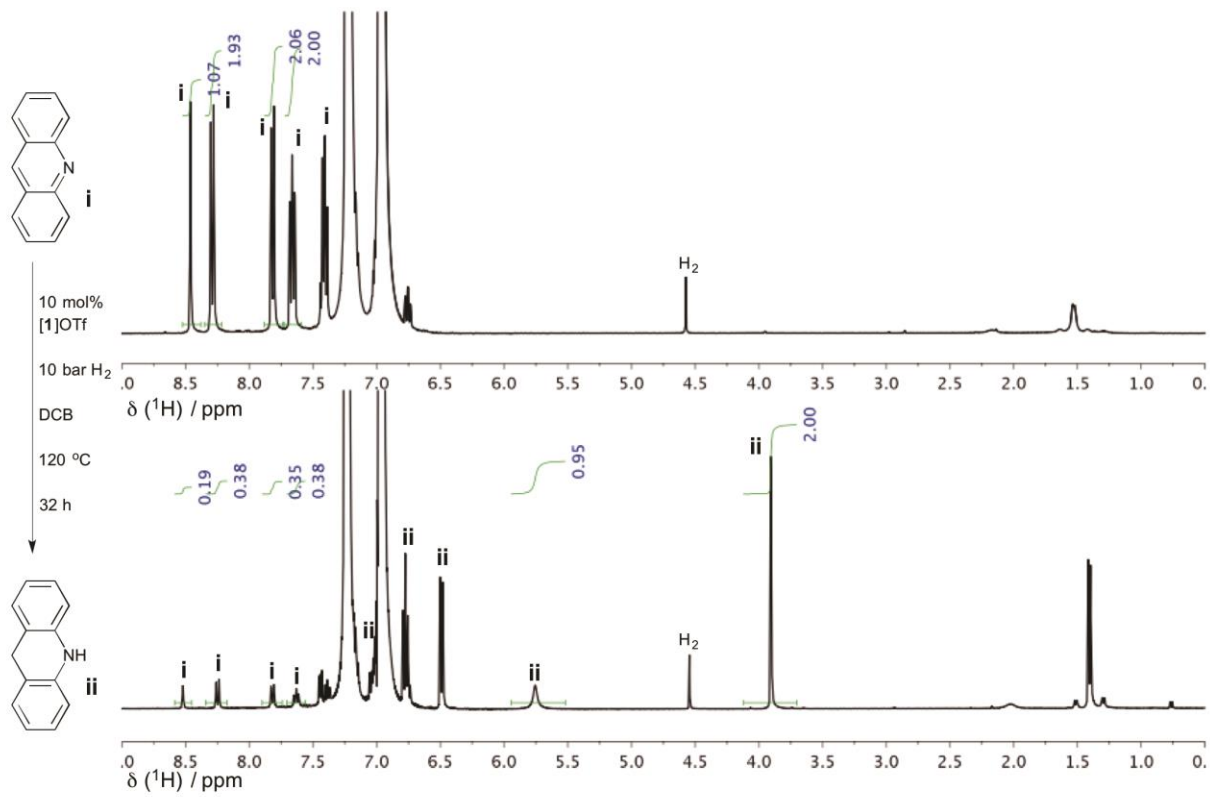

Figure S34 – ^{1}H NMR spectra for the hydrogenation of acridine

10.2. Hydrogenation of *n*-butyl acrylate

A solution of *n*-butyl acrylate (29 μL, 0.2 mmol) and collidine (2.6 μL, 0.02 mmol) in 1,2-dichlorobenzene (0.7 mL) was added to *i*Pr_3SnOTf ([**1**]OTf, 7.9 mg, 0.02 mmol) in a Wilmad high pressure NMR tube fitted with a PV-ANV PTFE valve. H_2 was admitted up to a pressure of 10 bar (at RT). The reaction mixture was heated in an oil bath to 120 ºC for 80 h. ^{1}H NMR spectroscopic analysis indicated 83 % conversion to *n*-butyl propanoate.

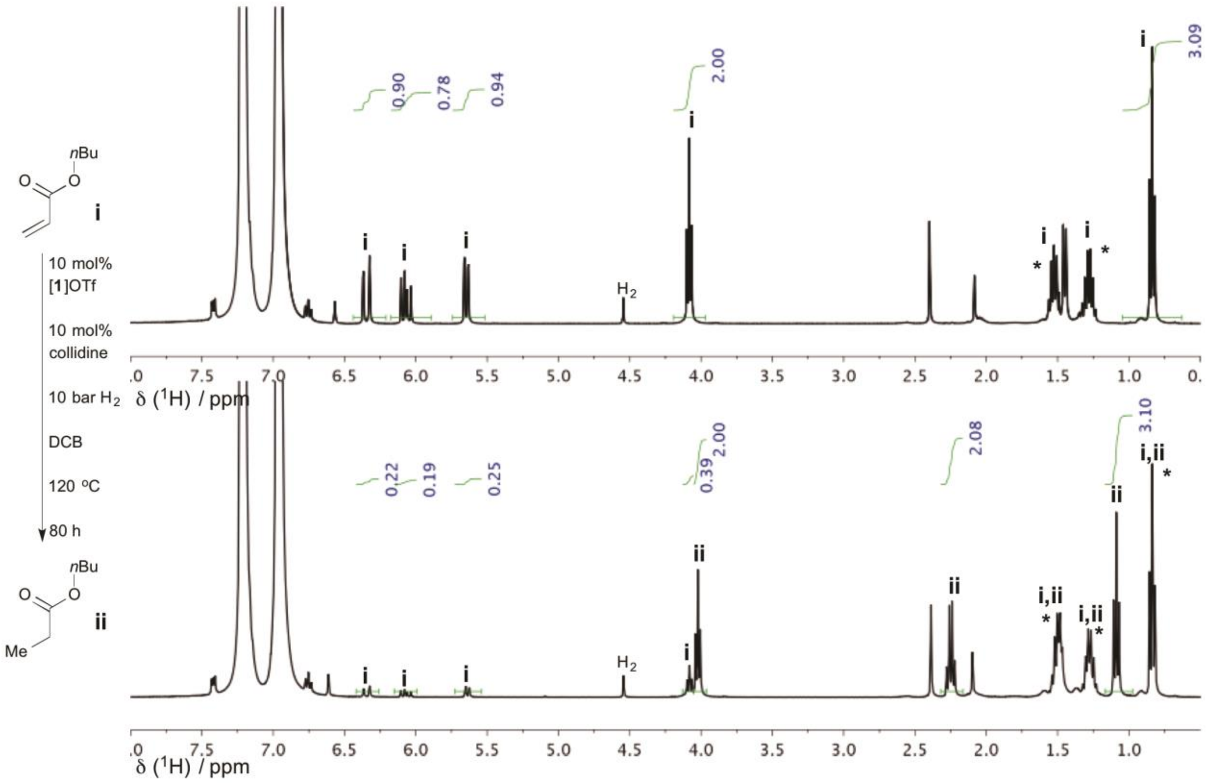

Figure S35 – ^{1}H NMR spectra for the hydrogenation of *n*-butyl acrylate (note that the peaks marked * overlap with each other, or with catalyst-based resonances)

10.3. Hydrogenation of 1-piperidino-1-cyclohexene

A solution of 1-piperidino-1-cyclohexene (33.1 mg, 0.2 mmol) and collidine (2.6 μL, 0.02 mmol) in 1,2-dichlorobenzene (0.7 mL) was added to iPr$_3$SnOTf ([**1**]OTf, 7.9 mg, 0.02 mmol) in a Wilmad high pressure NMR tube fitted with a PV-ANV PTFE valve. H_2 was admitted up to a pressure of 10 bar (at RT). The reaction mixture was heated in an oil bath to 120 °C for 4 h. ^{1}H NMR spectroscopic analysis indicated >99 % conversion to 1-cyclohexyl piperidine based on consumption of starting material.

To confirm the identity of the product, an authentic sample of 1-cyclohexyl piperidine (37 μL, 0.2 mmol) was combined with collidine (2.6 μL, 0.02 mmol) and [**1**]OTf (7.9 mg, 0.02 mmol) in 1,2-dichlorobenzene (0.7 mL), and H_2 was admitted up to a pressure of 10 bar (at RT). The ^{1}H NMR spectrum for this mixture closely matched that for the hydrogenation reaction.

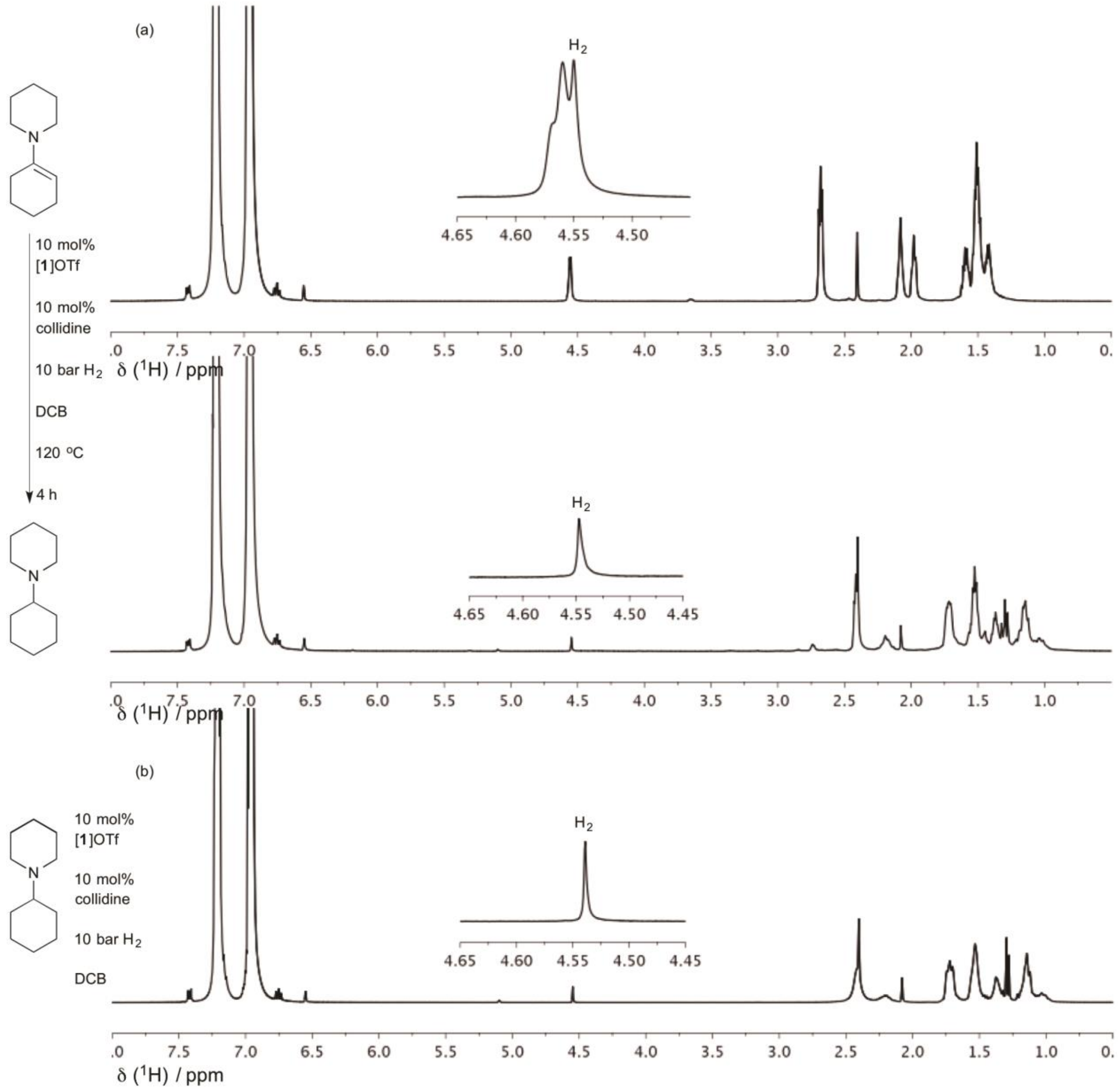

Figure S36 – ^{1}H NMR spectra for the hydrogenation of 1-piperidino-1-cyclohexene (a), and an independently-prepared sample of 1-cyclohexyl piperidine exposed to the catalytic mixture (b).

11. References

[1] J. Barluenga, A. Jiménez-Aquino, F. Aznar, C. Valdés, *J. Am. Chem. Soc.* **2009**, *131*, 4031-4041.

[2] J. J. M. Weemers, F. D. Sypaseuth, P. S. Bäuerlein, W. N. P. van der Graaff, I. A. W. Filot, M. Lutz, C. Müller, *Eur. J. Org. Chem.* **2014**, 350-362.

[3] K. Kloc, E. Kubicz, J. Mlochowski, L. Syper, *Synthesis* **1987**, 1084-1087.

[4] W. Kitching, H. A. Oisowy, G. M. Drew, *Organometallics* **1982**, *1*, 1244-1246.

[5] G. C. Welch, L. Cabrera, P. A. Chase, E. Hollink, J. D. Masuda, P. Wei, D. W. Stephan, *Dalton Trans.* **2007**, 3407-3414.

[6] M. A. Beckett, G. C. Strickland, J. R. Holland, K. S. Varma, *Polymer* **1996**, *37*, 4629-4631.

[7] A. E. Ashley, T. J. Herrington, G. G. Wildgoose, H. Zaher, A. L. Thompson, N. H. Rees, T. Krämer, D. O'Hare, *J. Am. Chem. Soc.* **2011**, *133*, 14727-14740.

[8] A. G. Davies, J. P. Goddard, M. B. Hursthouse, N. P. C. Walker, *J. Chem. Soc. Chem. Commun.* **1983**, 597-598.
